# Long noncoding RNA PANDA and scaffold-attachment-factor SAFA control senescence entry and exit

**DOI:** 10.1038/ncomms6323

**Published:** 2014-11-19

**Authors:** Pavan Kumar Puvvula, Rohini Devi Desetty, Pascal Pineau, Agnés Marchio, Anne Moon, Anne Dejean, Oliver Bischof

**Affiliations:** 1 Department of Pediatrics, University of Utah, Salt Lake City, Utah 84102, USA; 2 Weis Center for Research, Geisinger Clinic, Danville, Pennsylvania 17822, USA; 3 Institut Pasteur, Laboratory of Nuclear Organization and Oncogenesis, F-75015 Paris, France; 4 INSERM, U993, F-75015 Paris, France; 5 Equipe Labellisée Ligue Nationale Contre le Cancer, F-75015 Paris, France

## Abstract

Cellular senescence is a stable cell cycle arrest that limits the proliferation of pre-cancerous cells. Here we demonstrate that scaffold-attachment-factor A (SAFA) and the long noncoding RNA PANDA differentially interact with polycomb repressive complexes (PRC1 and PRC2) and the transcription factor NF-YA to either promote or suppress senescence. In proliferating cells, SAFA and PANDA recruit PRC complexes to repress the transcription of senescence-promoting genes. Conversely, the loss of SAFA–PANDA–PRC interactions allows expression of the senescence programme. Accordingly, we find that depleting either SAFA or PANDA in proliferating cells induces senescence. However, in senescent cells where PANDA sequesters transcription factor NF-YA and limits the expression of NF-YA-E2F-coregulated proliferation-promoting genes, PANDA depletion leads to an exit from senescence. Together, our results demonstrate that PANDA confines cells to their existing proliferative state and that modulating its level of expression can cause entry or exit from senescence.

## Abstract (web summary)

The gene-regulatory circuits that establish and maintain senescence remain incompletely understood. Here, the authors show that the long noncoding RNA PANDA and scaffold-attachment-factor A (SAFA) regulate entry and exit from senescence through context-specific interactions with PRC 1/2 and the transcription factor NF-YA.

## Graphical abstract

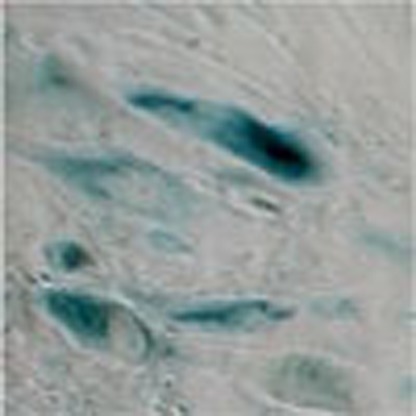

Cellular senescence is a proliferative arrest triggered by potentially cancer-causing events and it thus limits the outgrowth of pre-cancerous cells^1^. The gene-regulatory programme that initiates and maintains this phenotype remains by-and-large elusive but generally involves the stable repression of proliferation-promoting genes regulated by the retinoblastoma/E2F transcription factor complex^2^. Transcriptional regulation of E2F target genes is a highly complex process involving cooperation of E2F transcription factors with a large array of other promoter-specific transcription factors like CCAAT binding factor NF-YA^3^,^4^ or co-repressors including components of the RNAi machinery and histone methyl transferases among others^5^. Senescent cells also strongly de-repress senescence-enforcing genes such as *CDKN1A (*alias *p21)* and *CDKN2A (*alias *p16)*, as well as a plethora of secretory factors and their receptors (for example, *IL8, BMP2, WNT* or *MMP3)*, all of which are pivotal for the proper and timely execution of the senescence programme^1^. Many of the above genes are repressed in proliferating cells by polycomb repressor complexes (PRC) and accordingly, functional ablation of certain polycomb group (PcG) proteins induces a senescence arrest^6^,^7^,^8^,^9^.

PcG proteins broadly assemble in two different multi-protein complexes: PRC1 and PRC2. Core components of PRC1 include the ubiquitin ligase RING1A/B, BMI1 (PCGF4), NSCP1 (PCGF1) and MEL18, whereas those of PRC2 include histone methyl transferase EZH2, EED and SUZ12, though it becomes increasingly clear that PRC complexes are heterogeneous with variable composition^10^. PRC1 catalyses mono-ubiquitination of lysine 119 on histone H2A (H2AK119_ub1_), and together with PRC2, which catalyses trimethylation of lysine 27 on histone H3 (H3K27_me3_), it creates an inactive chromatin environment to control cell-fate decisions^10^,^11^. A central question is how PcG proteins are recruited to their cognate gene targets given their lack of an inherent DNA binding activity. Several mechanisms have been proposed^12^,^13^,^14^,^15^ and there is an increasing evidence that gene-selective recruitment may be driven by the varying composition of PRCs^16^. More recently, nascent^17^ and long noncoding (lnc) RNAs have been shown to direct PRCs to site-selected targets. LncRNAs modulate gene expression in multiple ways but few have been fully functionally characterized^18^. They may work as guides or modular chromatin scaffolds to recruit co-repressors or co-activators but may also operate as decoys as is the case for lncRNA PANDA, a lncRNA coregulated together with *CDKN1A* by tumour suppressor protein p53. For example, PANDA transiently sequesters transcription factor NF-YA to suppress its pro-apoptotic function during a DNA damage response^19^.

SAFA is a very abundant multimodular nuclear protein that is able to bind DNA and RNA including several classes of noncoding RNA^20^,^21^,^22^. It is involved in various transcriptional and posttranscriptional processes. Notably, it was shown to be instrumental for the deposition of lncRNA Xist and PRC2-mediated silent chromatin mark H3K27_me3_ at the inactive X chromosome^20^,^23^,^24^. Yet, the precise role that SAFA has in most of these processes remains to be determined. We hypothesized that because of its intrinsic DNA- and RNA-binding activity, SAFA would naturally lend itself to operate as an adaptor molecule for DNA–RNA-protein interactions to regulate gene expression and given its implication in PRC-mediated H3K27_me3_ deposition to regulate cell-fate decisions. Here we report a direct physical and functional association between SAFA and lncRNA PANDA with PRC1, PRC2 and transcription factor NF-YA in a cell-fate dependent manner. We provide evidence that SAFA and PANDA control chromatin access of PRCs and NF-YA to pro-senescence and pro-proliferation target genes to regulate the cell cycle arrest associated with senescence.

## Results

### SAFA depletion leads to the onset of cellular senescence

**Figure 1:**
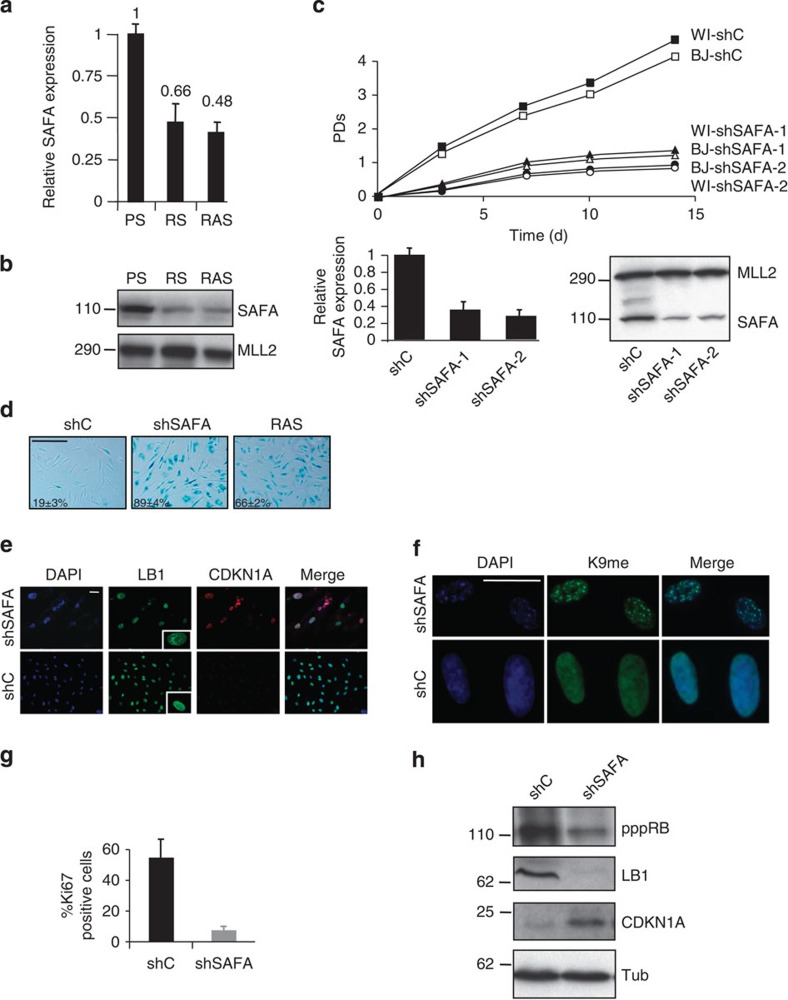
SAFA repression actively contributes to senescence entry. (**a**) Relative quantification of SAFA expression by qRT–PCR (*P*≤0.05) and (**b**) representative (*n*=3) western blot analysis comparing BJ fibroblasts undergoing replicative (RS) or RAS^V12^ (RAS) senescence to pre-senescent proliferating (PS) BJ fibroblasts. MLL2 served as loading control. s.d. of three independent qRT–PCR experiments each in quadruplicates is indicated; *P*≤0.05, *t*-test. (**c**–**h**) Depletion of SAFA induces a senescence response in fibroblasts. (**c**) Representative proliferation curves of biological replicates (*n*=3) of BJ and WI38 fibroblasts infected either with pLKO.1-shScramble (shC), pLKO.1-shSAFA-1 or pLKO.1-shSAFA-2. After day 2 of drug selection, the number of population doublings (PDs) was determined over the indicated period of time. Day 0 is the first day after selection. PDs for each time point are the mean value of triplicates. Also, shown is the expression of SAFA using qRT–PCR (lower left panel) and representative (*n*=3) western blot analysis (lower right panel). MLL2 served as loading control. s.d. of three independent experiments is indicated for qRT–PCR; *P*≤0.05, *t*-test. (**d**) Representative micrographs showing BJ fibroblast cell morphology and SABG staining plus percentage of SABG-positive cells for BJ fibroblasts transduced either with pLKO.1-shC, pLKO.1-shSAFA-1 (shSAFA) or pBABE-RAS^V12^ (RAS), the latter serving as positive control; scale bar, 400 μM. s.d. of three independent experiments (*n*=3) is indicated; *P*≤0.05. (**e**) Indirect immunostaining for lamin B1 (LB1), CDKN1A and (**f**) H3K9_me3_ (K9me) to visualize senescence-associated heterochromatin foci. Note the dysmorphic nuclear shape in the inset of Fig. 3e; scale bar, 20 μM. (**g**) Bar chart for cells staining positive for proliferation marker Ki67 at day 6 post selection. Error bars represent the s.d. of three independent experiments; *P*≤0.05, *t*-test; (*n*=200 cells per count). (**h**) Immunoblot analysis for hyper-phosphorylated RB (pppRB), LB1 and CDKN1A. Tubulin (Tub) served as loading control.

We previously performed comprehensive genome-wide gene expression profiling to identify genes that are differentially regulated in cellular senescence^5^,^25^,^26^. A number of genes have been shown to promote senescence upon loss of function^7^,^25^,^27^,^28^. Among the genes that were consistently downregulated by at least 1.5-fold (*P*=≪0.01) in cells undergoing senescence was the scaffold-attachment-factor A (SAFA alias hnRNPU or SP120). To confirm our microarray results, we performed quantitative real-time PCR (qRT–PCR) and immunoblot analyses. Senescent cells induced either by replicative exhaustion (that is, replicative senescent, RS) or oncogenic Ras^V12^ (RAS) displayed a reduction in SAFA transcript (Fig. 1a) and protein levels (Fig. 1b). Thus, the downregulation of SAFA gene expression coincides with the onset of senescence induced by two different signals. To establish that a decreasing pool of SAFA directly leads to senescence, we targeted its expression by short hairpin interference in two normal, primary human diploid fibroblast strains, BJ (neonatal foreskin) and WI38 (fetal lung), using a scrambled control (shC) and two individual knock-down constructs against SAFA (shSAFA-1 and shSAFA-2). BJ and WI38 fibroblasts represent the two extremes in a spectrum of p53 or p53/Rb/CDKN2A-dependent senescence with BJ fibroblast senescence requiring solely the p53- (but not CDKN2A/Rb pathway) and WI38 senescence utilizing both p53 and CDKN2A/Rb tumour suppressor pathway(s)^29^. BJ and WI38 fibroblasts depleted for SAFA became fully senescent after 4–6 days as judged by a stable proliferative arrest (Fig. 1c; data for WI38 shown only for proliferation curves), an increase in cells staining positive for senescence-associated beta-galactosidase (SABG) (Fig. 1d), CDKN1A (Fig. 1e) and senescence-associated heterochromatin foci (Fig. 1f), a decrease in cells staining positive for proliferation marker Ki67 (Fig. 1g), the appearance of dysmorphic nuclei as measured by lamin B1 (LB1) indirect immunostaining (Fig. 1e) and repression of E2F-responsive genes such as *AURKA*, *BRCA1*, *CCNB1*, *CCNA2*, *CDC2*, *CDC25C*, *CDK1*, *E2F1*, *MCM3* and *TK1* (Supplementary Fig. 1). In addition, immunoblotting showed a decrease in Rb phosphorylation and LB1 protein levels (the latter being congruent with the observed malformation of the nuclear envelope; see Fig. 1e) accompanied by a simultaneous increase in CDKN1A protein levels in SAFA-depleted (shSAFA) compared with control (shC) cells (Fig. 1h). Together, these results argue that silencing of the SAFA expression is not merely associated with the senescence phenotype but actively contributes to it.

### SAFA is a component of PRC1 and PRC2

**Figure 2:**
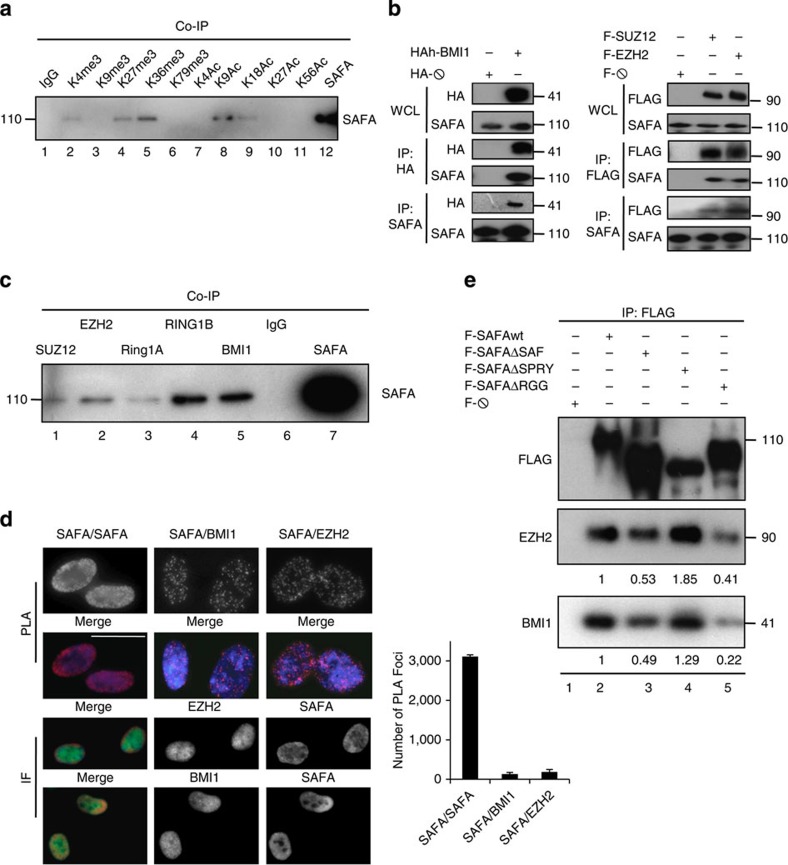
SAFA interacts PcG proteins *in vivo*. (**a**) Representative global SAFA binding to active and inactive histone marks as detected by histone association assay. Proliferating BJ fibroblasts were crosslinked by PFA, sonicated and lysed. Euchromatin- and heterochromatin-bound SAFA was identified by co-immunoprecipitation (Co-IP) using indicated antibodies. Immunoprecipitates were western-blotted with SAFA-specific antibody; *n*=3. (**b**) Representative immunoprecipitation (IP) with antibodies to HA, FLAG and SAFA in cellular lysates prepared from HEK293 cells overexpressing PcG proteins HA-BMI1 (left panel), FLAG-SUZ12 and FLAG-EZH2 (right panel) or empty vectors. Immunocomplexes were analysed by western blot with antibodies specific for HA, FLAG and SAFA. WCL, whole cell lysate, 2.5% of total used for IP; *n*=3. (**c**) Representative co-immunoprecipitation (Co-IP) of endogenous SAFA with PcG proteins SUZ12, EZH2, RING1A, RING1B and BMI1, in lysates of proliferating BJ fibroblasts using indicated antibodies or IgG. Immunoprecipitates were western-blotted with antibody to SAFA; *n*=3. (**d**) Representative proximity-ligation assay (PLA) (upper panel) and classical indirect immunostaining (lower panel) for SAFA and PcG proteins BMI1 and EZH2 in proliferating BJ fibroblasts. Each dot represents a positive protein–protein interaction in PLA. Also shown is the quantification of PLA interactions (right bar chart). DAPI was used to counterstain nuclear DNA in merged images. Scale bar, 20 μM. Error bars represent the s.d. of three independent experiments; *P*≤0.05, *t*-test (*n*=100 cells per count). (**e**) DNA- (SAF) and RNA (RGG)-binding domain are important for interaction between SAFA and PcG proteins. Representative immunoprecipitation (IP) with antibody to FLAG in cellular lysates prepared from HEK293 cells overexpressing wild-type (wt) and mutant (Δ) SAFA proteins or empty vector. Immunocomplexes were analysed by western blot with antibodies specific for FLAG, BMI1 and EZH2. Ratios were calculated densitometrically; *n*=3.

As a first step towards an understanding as to how SAFA might be involved in senescence, we examined in an unbiased fashion whether SAFA has a predilection for certain chromatin states by performing a histone association assay^30^. All antibodies directed against posttranslational histone modifications precipitated their respective targets efficiently (Supplementary Fig. 2a) and we detected interactions between SAFA and active chromatin marks histone H3 trimethylated on lysine 4 (H3K4_me3_), histone H3 trimethylated on lysine 36 (H3K36_me3_), histone H3 acetylated on lysine 9 (H3K9_ac_) and histone H3 acetylated on lysine 18 (H3K18_ac_; Fig. 2a, lanes 2, 5, 8 and 9). We also observed a robust binding of SAFA to the PRC2-catalysed repressive histone mark H3K27_me3_ (Fig. 2a, lane 4) and because PRC components are essential for the timely and proper execution of senescence^6^,^7^,^8^,^9^, we tested the possibility that SAFA cooperates with PcG proteins first by performing co-immunoprecipitations in cells ectopically expressing either HA-tagged PRC1 component BMI1 (alias PCGF4), FLAG-tagged PRC2 core components EZH2 and SUZ12 or corresponding empty vectors. Overexpressed HA-BMI1 (Fig. 2b, left panels), FLAG-EZH2, FLAG-SUZ12 (Fig. 2b, right panels) and endogenous SAFA (Fig. 2b, left and right panels) reciprocally co-immunoprecipitated each other demonstrating that SAFA interacts with these PcG proteins *in vivo*. To extend the physiological relevance for these interactions, we carried out co-immunoprecipitations between endogenous SAFA and PRC1 core subunits BMI1, RING1A and RING1B, as well as PRC2 core subunits EZH2 and SUZ12 in whole cell lysates prepared from low passage, proliferating BJ fibroblasts. As depicted in Fig. 2c and Supplementary Fig. 2b, a sub-fraction of SAFA specifically co-immunoprecipitated with all tested PcG proteins (compare lanes 1, 2, 3, 4, 5 with lanes 6 and 7). By contrast, SAFA did not interact with NSCP1 (alias PCGF1, Supplementary Fig. 2c), which is the distinct determinant of a non-BMI1 containing PRC1 complex^16^. These results thus suggest that SAFA is part of a PRC1 complex that includes BMI1 and Ring1A/B but not NSCP1, which is congruent with the finding that PRC1 subcomplexes are distinguished by different PCGF family members^16^. We then used proximity-ligation assay (PLA) to visualize and quantify endogenous SAFA–SAFA, SAFA–BMI1 and SAFA–EZH2 interactions in proliferating fibroblasts^31^. As displayed in Fig. 2d (upper panels PLA), we detected on average ~3,100 SAFA–SAFA, ~138 SAFA–BMI1 and ~186 SAFA–EZH2 distinct PLA-foci/interactions, thus, only a small fraction of endogenous SAFA molecules is available for PRC interactions in a nucleus, which is corroborated further by classical indirect immunofluorescence analysis of SAFA/BMI1 and SAFA/EZH2 nuclear distribution (Fig. 2d lower panels IF). Technical and biological controls showed that PLA was highly sensitive and very specific for interactions between endogenous proteins (Supplementary Fig. 2d). Next, we dissected which SAFA domains are necessary for optimal binding to PcG proteins. To this end, we assayed cell lysates from cells overexpressing FLAG-tagged SAFAwt (wild-type), SAFAΔSAF (DNA-binding-deficient mutant), SAFAΔSPRY (mutant SPla and RYanodine receptor domain of unknown function) or SAFAΔRGG (RNA-binding-deficient mutant) proteins^23^ (Supplementary Fig. 2e) by co-immunoprecipitations with antibody to FLAG. The bound proteins were analysed by western blotting with antibodies against BMI1 and EZH2 serving as proxies for PRC1 and PRC2 core subunits. Both proteins co-immunoprecipitated very efficiently with SAFAwt and SAFAΔSPRY proteins (Fig. 2e, compare lanes 2 and 4). By contrast, the association of BMI1 and EZH2 with SAFAΔSAF or SAFAΔRGG was reduced by ~2-5-fold (Fig. 2e, compare lane 2 with lanes 3 and 5). Together, these results provide ample evidence that SAFA can interact with BMI1–PRC1 and EZH2/SUZ12–PRC2 complexes and that its DNA as well as RNA-binding domains contribute to optimal complex formation.

### SAFA regulates expression of PRC target genes

**Figure 3:**
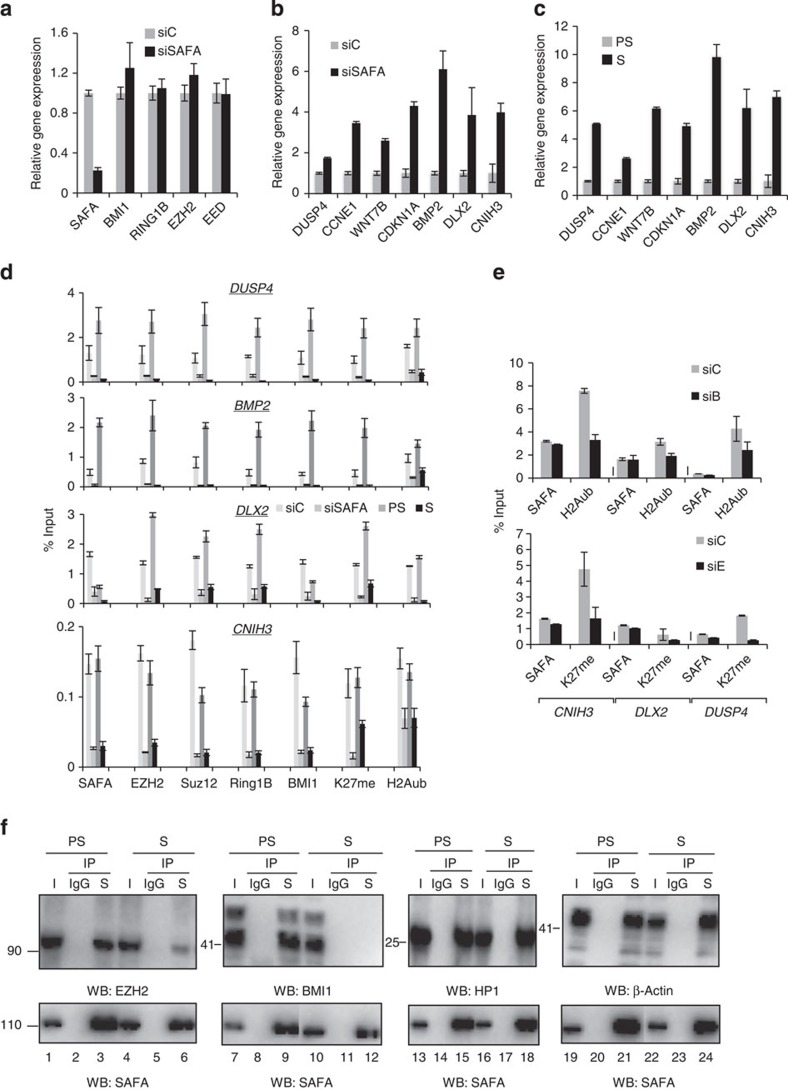
SAFA regulates expression of pro-senescence genes via recruitment of polycomb repressor complexes PRC1 and PRC2. (**a**,**b**) Pre-senescent proliferating BJ fibroblasts were transiently transfected with siScramble control (siC) or siSAFA. qRT–PCR on total RNA prepared from respective samples at day 2 of siRNA treatment for indicated genes. Error bars represent the s.d. of three independent experiments; *P*≤0.05, *t*-test. (**c**) qRT–PCR on total RNA prepared from pre-senescent proliferating (PS) and RAS-senescent (S) BJ fibroblasts for indicated genes. Error bars represent the s.d. of three independent experiments; *P*≤0.05, *t*-test. (**d**) SAFA, EZH2, SUZ12, RING1B, BMI1, H3K27_me3_ (K27_me_) and H2AK119_Ub1_ (H2A_Ub_) qChIP–PCR for indicated genes was performed in proliferating BJ fibroblasts at day 2 of siC and siSAFA treatment or in pre-senescent proliferating (PS) and RAS-senescent (S) BJ fibroblasts. (*n*=3); *P*≤0.05. (**e**) H2AK119_Ub1_ (H2A_Ub_) or H3K27_me3_ (K27_me_) and SAFA-qChIP–PCR for indicated gene promoters was performed at day 2 of siControl (siC), siBMI1 (siB) or siEZH2 (siE) treatment in pre-senescent proliferating (PS) BJ fibroblasts. *n*=3; *P*≤0.05, *t*-test. (**f**) Representative co-immunoprecipitation (IP) of endogenous SAFA (S) with EZH2, BMI1 as well as known interactors HP1 and β-actin in EtOH-treated pre-senescent proliferating (PS) and 4OHT-induced ER:Ras^V12^ senescent (S) BJ fibroblasts at day 2 post induction with tamoxifen using IgG and an antibody specific to SAFA (S). western blot analysis with antibodies to SAFA (lower panel), EZH2, BMI1, HP1α and β-actin (upper panel); *n*=3.

We next sought to determine the functional relevance of the interaction between SAFA and PRC complexes. A number of PRC response genes, notably *CDKN1A*, *CDKN2A*, *IL6R, IL8, JUNB, DUSP4* and *BMP2*, were shown to encode important pro-senescence factors^32^,^33^,^34^,^35^,^36^,^37^. Comparison of our list of genes upregulated in senescence^5^ to two published lists of potential human PRC target genes^38^,^39^ showed that ~7% of upregulated genes in senescence constitute potential PRC targets and that ~24% of potential PRC targets are de-repressed by at least 2-fold in senescence (Supplementary Fig. 3a and Supplementary Data). On the basis of our interaction study, we hypothesized that SAFA is required for proper transcriptional repression of PRC target genes. If so, silencing of SAFA expression should result in de-repression of PRC target genes. To test this hypothesis, we first assayed total RNA prepared from control siRNA (siC), siSAFA (siS)-, siBMI1 (siB)-, siEZH2 (siE)-transfected cells as well as from pre-senescent proliferating (PS) and RAS-senescent (S) BJ fibroblasts after 48 h of siRNA treatment by qRT–PCR for the expression status of selected PRC target genes upregulated in senescence (that is, *DUSP4, CCNE1, WNT7B, CDKN1A, BMP2, DLX2* and *CNIH3)*. SAFA expression was markedly decreased by a pool of two validated siRNAs^40^ whereas the expression of PRC core components BMI1, RING1B, EZH2 and EED was unaffected by the transient knock-down treatment (Fig. 3a and Supplementary Fig. 3b). This analysis also revealed the de-repression of the selected PRC response genes by 2 to 9-fold in SAFA- (Fig. 3b), BMI1-, EZH2-depleted (Supplementary Fig. 3c,d) and in senescent cells (Fig. 3c) when compared with respective controls. The observed de-repression was positively correlated with a strongly decreased occupancy of SAFA, EZH2, SUZ12, BMI1, RING1B, as well as PRC-catalysed inactive chromatin marks H3K27_me3_ and H2AK119_ub1_ at respective PRC target gene promoters as determined by chromatin immunoprecipitation followed by quantitative real-time PCR (qChIP–PCR, Fig. 3d and Supplementary Fig. 3e). Since transient SAFA silencing did not affect BMI1, RING1B, EED or Ezh2 levels, the most plausible explanation for the decreased target gene occupancy of PRC components is that SAFA contributes to their recruitment in proliferating cells. Next, we examined whether the genomic binding of SAFA depends on PcG proteins by SAFA-qChIP–PCR in BMI1- and EZH2-depleted cells. As summarized in Fig. 3e, we found that neither silencing of BMI1 (siB) (upper panel) nor EZH2 (siE)(lower panel) impacted on SAFA presence at selected target genes. By contrast, BMI1 depletion affected H2AK119_ub1_ (upper panel) and EZH2 depletion H3K27_me3_ (lower panel) deposition at the interrogated targets. We thus conclude that SAFA gene target binding does not require intact BMI1–PRC1 and EZH2–PRC2 complexes. Congruent with the above results, we found the interaction between SAFA and BMI1, as well as SAFA and EZH2 to be impaired in cells undergoing oncogene-induced senescence compared with controls already at a time point (day 2) when cells are in transit between the initial mitotic (day 1) and the senescence phase (day 6 and beyond)^25^,^41^ and EZH2 and BMI1 levels are not yet diminished as observed in the senescence phase (Fig. 3f; compare lane 1 with 4, 3 with 6, 7 with 10 and 9 with 12; Supplementary Fig. 3f,g). By contrast, the interaction between SAFA and two known interactors, HP1 (ref. 40) and β-actin^42^, was unperturbed (Fig. 3f; compare lane 13 with 16, 15 with 18, 19 with 22 and 21 with 24) as was the binding between EZH2 and SUZ12, as well as BMI1 and RING1A in SAFA-silenced and senescent cells when compared with controls (Supplementary Fig. 3h). Next, we studied the effects of SAFA downregulation on global H3K27_me3_ and H2AK119_ub1_ levels. Knock-down of SAFA neither significantly affected the global cellular levels of H3K27_me3_ nor H2AK119_ub1_ congruent with published results^23^ (Supplementary Fig. 3i). This observation is consistent with the idea that interference with SAFA function results in only gene-specific changes in H3K27_me3_ or H2AK119_ub1_. Collectively, the above results support the notion that SAFA is required for efficient recruitment of BMI1–PRC1 and –PRC2 complexes to select senescence target genes to silence their expression in pre-senescent, proliferating cells. By contrast, in SAFA-depleted cells and in cells undergoing senescence, the BMI1–PRC1 EZH2–PRC2 complexes are displaced from chromatin, and these genes become de-repressed.

### SAFA and PRCs negatively regulate *CDKN1A* and lncRNA *PANDA*

**Figure 4:**
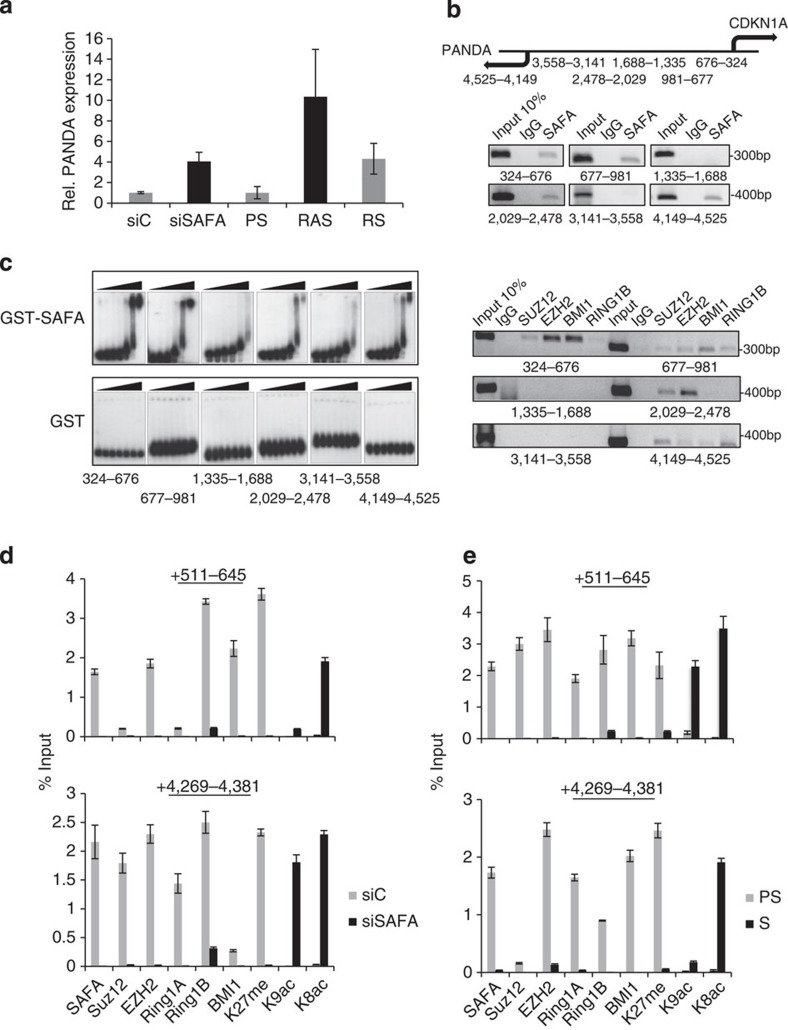
SAFA and PRCs are negative co-regulators of pro-senescence factor *CDKN1A* and lncRNA PANDA. (**a**) PANDA expression as determined by qRT–PCR in siScramble control (siC), siSAFA-treated and pre-senescent proliferating (PS), RAS-senescent and replicative senescent (RS) BJ fibroblasts. Experiment was performed in triplicates and repeated three times; s.d. is indicated; *P*≤0.05; rel., relative. (**b**) Top: schematic diagram of transcriptional start site (TSS) of *CDKN1A* and *PANDA* (arrows). Shown are also the promoter regions used for DNA amplification of ChIP material. Bottom: IgG, SAFA (upper panel) and IgG, SUZ12, EZH2, BMI1 and RING1B (lower panel) semi-quantitative ChIP-PCR of indicated promoter regions. (**c**) Representative electrophoretic mobility shift assay (EMSA) for indicated promoter regions using 10, 20, 40, 80, 160, 320 and 500 ng recombinant GST-SAFA and GST proteins in 25 μl reaction volume; *n*=3. (**d**,**e**). SAFA, SUZ12, EZH2, BMI1, RING1B, H3K27_me3_ (K27_me_), H3K9_ac_ (K9_ac_) and H4K8_ac_ (K8_ac_) qChIP–PCR of indicated promoter regions in (**d**) siScramble control (siC) and siSAFA-treated proliferating BJ fibroblasts and (**e**) pre-senescent proliferating (PS) and RAS-senescent (S) BJ fibroblasts. *n*=3; *P*≤0.05, *t*-test.

*CDKN1A* is a canonical transcriptional target gene of p53, PRC complexes^33^ and SAFA (see Fig. 3b). Its transcription is strongly induced in cells undergoing senescence and it has a critical role in the establishment of the senescence phenotype^1^. Recently, P53 was shown to positively co-regulate *CDKN1A* and lncRNA *PANDA* (hereafter termed PANDA) expression. The transcriptional start site (TSS) of *PANDA* was mapped ~4.5 kbp upstream of the *CDKN1A* TSS and ~2.5 kbp upstream of the major P53 response element^19^. Because SAFA is a repressor of *CDKN1A* in pre-senescent proliferating cells (see Fig. 3b), we asked whether SAFA also represses *PANDA* expression in these cells. Indeed, *PANDA* transcript abundance was robustly increased ~4-fold in SAFA (siS)-depleted cells when compared with respective controls (siC) and ~4–10-fold in RAS and RS cells (Fig. 4a). To map SAFA and PRC binding sites within the *CDKN1A* promoter more precisely, we performed SAFA-ChIP–PCR in nuclear lysates prepared from pre-senescent proliferating BJ fibroblasts with six primer pairs amplifying genomic regions that are located +324–676, +677–981, +1,335–1,688, +2,029–2,478, +3,141–3,558 and +4,149–4,525 bp upstream of the *CDKN1A* TSS. Apart from region +3,141–3,558, all other regions were identified previously as bona fide P53 promoter binding sites^43^,^44^ with the +4,149–4,525 region protruding into the PANDA gene. While SAFA and PcG proteins SUZ12, EZH2, BMI1 and RING1B co-occupied proximal *CDKN1A* promoter regions +324–676, +677–981 and distal region +4,149–4,525, only SAFA and PRC2 core components SUZ12 and EZH2 were enriched on the distal region +2,029–2,478, whereas regions +1,335–1,688 and +3,141–3,558 were negative for any binding (Fig. 4b). To substantiate the direct physical association between SAFA and the ChIP-identified *CDKN1A* promoter regions, gelshift experiments were performed with purified recombinant GST-SAFA and GST proteins (Supplementary Fig. 4). This analysis illustrated that GST-SAFA, but not GST, has the potential to bind specifically and autonomously to regions +324–676, +677–981, +4,149–4,525 and +2,029–2,478; but only weakly to +1,335–1,688 and +3,141–3,558, thus, confirming our above-shown ChIP results (Fig. 4c). Next, we examined SAFA, EZH2, SUZ12, BMI1, RING1A/B, repressive chromatin marks H3K27_me3_ and H3K9_me3_, as well as active chromatin marks H3K9_ac_ and H4K8_ac_ occupancies in SAFA-depleted and senescent cells within the *CDKN1A* promoter region +324–676 and the *PANDA* protruding region +4,149–4,525, respectively using SAFA-qChIP–PCR. Congruent with the above results, showing that SAFA and PRCs work as repressors of *CDKN1A* and *PANDA*, we found that, both in SAFA-silenced (siSAFA) (Fig. 4d) and in RAS-senescent cells (S) (Fig. 4e), the presence of SAFA, EZH2, SUZ12, BMI1, RING1A/B, H3K27_me3_ and H3K9_me3_ was markedly decreased compared with controls (siC and PS, respectively), whereas the active chromatin marks H3K9_ac_ and H4K8_ac_ were enriched at the respective promoters. We conclude that SAFA and PRCs are negative co-regulators of *CDKN1A* and *PANDA* expression and that SAFA can associate autonomously with specific promoter regions. These data, thus, further corroborate the notion that SAFA constitutes a crucial factor regulating the access of PcG protein complexes to select senescence target genes in a sequence-specific fashion in pre-senescent proliferating cells. Finally, given the differential expression of PANDA in pre-senescent versus senescent cells, our data also point to a potential involvement of PANDA in senescence.

### PANDA and SAFA reinforce their interactions with BMI1

**Figure 5:**
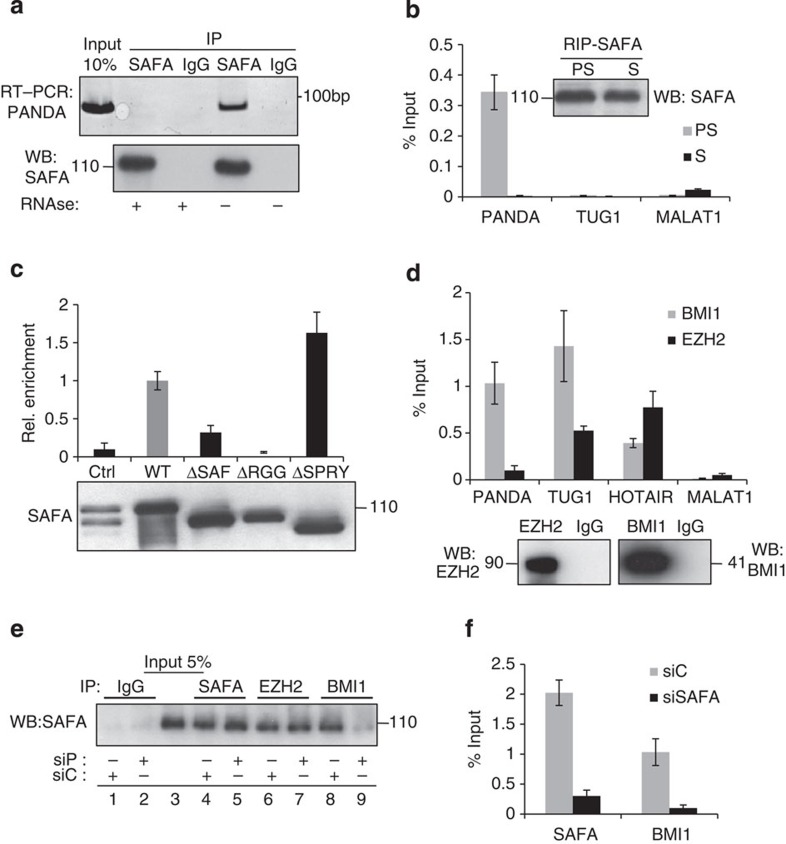
PANDA binds to SAFA and BMI1 and is critical for their interaction. (**a**) Representative (*n*=3) native RNA immunoprecipitation (RIP) of PANDA using antibody to SAFA or IgG in RNAse A-treated (+) or untreated (−) HEK293 cell lysates. Immunocomplexes were analysed for PANDA by RT–PCR and SAFA-specific antibody by immunoblot. (**b**) Native qRIP in lysates from presenesent proliferating (PS) and RAS-senescent (S) BJ fibroblasts using an antibody to SAFA. Precipitated RNA was analysed for PANDA as well as TUG1 and MALAT1 as controls by qRT–PCR and SAFA by immunoblot (inset). Error bars represent the s.d. of three independent experiments performed in triplicates; *P*≤0.05, *t*-test. (**c**) RNA- (RGG) and DNA- (SAF) binding domains of SAFA are important for PANDA association. Native RIP with antibody to FLAG in cellular lysates prepared from HEK293 cells overexpressing FLAG-tagged wild-type (wt) and mutant (Δ) SAFA proteins or empty vector (Ctrl). Immunocomplexes were analysed for PANDA by qRT–PCR and antibody to FLAG by immunoblot. Error bars represent the s.d. of three independent experiments; *P*≤0.05, *t*-test. (**d**) Native qRIP in lysates from pre-senescent proliferating BJ fibroblasts using antibodies specific to EZH2 and BMI1. Immunocomplexes were analysed for PANDA, TUG1, HOTAIR and MALAT1 by qRT–PCR and EZH2 or BMI1 by immunoblot. Error bars represent the s.d. of three independent experiments performed in triplicates; *P*≤0.05, *t*-test. (**e**) Representative co-immunoprecipitation (IP) of endogenous SAFA with EZH2 and BMI1 in lysates from siScramble control (siC) and siPANDA-treated BJ fibroblasts using indicated antibodies. Immunocomplexes were analysed by western blot with antibody to SAFA. (**f**) Native qRIP in lysates from siScramble control (siC) and siSAFA-treated proliferating BJ fibroblasts using antibodies to SAFA and BMI1. Immunocomplexes were analysed for PANDA by qRT–PCR. Error bars represent the s.d. of three independent experiments performed in triplicates; *P*≤0.05, *t*-test.

SAFA binds to many different classes of regulatory noncoding RNAs^22^ and its RGG domain is necessary for RNA interaction^21^. Moreover, lincRNA-p21 was shown to physically associate with the SAFA family member hnRNP-K to regulate transcription^45^. We therefore entertained the idea that SAFA and PANDA could physically and functionally interact to control gene expression. Indeed, endogenous PANDA RNA co-immunoprecipitated with SAFA in cellular lysates prepared from 293HEK cells (Fig. 5a). Importantly, the interaction was not detected when an isogenic IgG antibody was used and in RNAse-treated samples excluding the presence of contaminating DNA. We then extended this study to cell lysates prepared from pre-senescent proliferating (PS) and senescent (S) fibroblasts. Here, we detected a robust and specific interaction between SAFA and PANDA, but not other lncRNAs such as TUG1 or MALAT1, in pre-senescent proliferating but not senescent cells (Fig. 5b). qRT–PCR revealed that PANDA was present at ~130 copies per nucleus in proliferating and ~1,200 copies per nucleus in senescent fibroblasts (Supplementary Fig. 5a). These numbers are intriguing given that we detected about 130 SAFA–BMI1 and 180 SAFA–EZH2 interactions in proliferating cells (see Fig. 2c) indicating that PANDA may be primarily engaged in SAFA–BMI1 complexes. Next, we mapped the domains essential for this interaction by RIP in cells overexpressing SAFA FLAG-tagged wild-type and mutant constructs (see Fig. 2e and Supplementary Fig. 2e) using FLAG-specific antibody followed by qRT–PCR and immunoblot. This analysis demonstrated that SAFA lacking the RGG RNA-binding domain (ΔRGG) was completely deficient for PANDA binding, and SAFA lacking the SAF DNA-binding domain (ΔSAF) had strongly reduced binding for PANDA (~3-fold). By contrast, SAFA lacking the SPRY domain (ΔSPRY) showed slightly increased binding to PANDA by ~1.6-fold when compared with wild-type SAFA (Fig. 5c). Recently, lincRNAs were shown to interact with several chromatin regulatory complexes^45^. We thus sought to determine whether or not PANDA modulates the interaction between SAFA and PRC1 and PRC2 core components. Indeed, BMI1 efficiently co-immunopreciptated PANDA while EZH2 was negative for binding, the latter being in line with published results^19^ (Fig. 5d). By contrast two lncRNAs, TUG1 and HOTAIR, that were shown to interact with EZH2, were positive while MALAT1 was negative for binding^46^. Remarkably, the interaction between SAFA and BMI1 was specifically perturbed by a pool of two validated PANDA siRNAs^19^ (siP), but not by the respective siControl (siC; Fig. 5e, compare lanes 8 and 9), whereas the binding between SAFA and EZH2 was unaffected under both conditions (Fig. 5e, compare lanes 6 and 7). Importantly, transient PANDA depletion did not alter expression levels of SAFA, EZH2 and BMI1 as shown by qRT–PCR analysis (Supplementary Fig. 5b) and the equal amounts of these proteins in siControl and siPANDA-treated cell extracts (Fig. 5e for SAFA, compare lanes 4 and 5; Supplementary Fig. 5c for EZH2 and BMI1). We next tested the effect of SAFA depletion on BMI1–PANDA interaction. As shown in Fig. 5f, complex formation between BMI1 and PANDA was strongly reduced in SAFA-silenced (siSAFA) cells compared with controls (siC). This is consistent with our additional finding showing that the RNA-binding domain of SAFA is critical for optimal binding both to BMI1 as well as PANDA (see Figs 2e and 5c). We also note that the interaction between SAFA and other PcG proteins including SUZ12, RING1A and RING1B was entirely RNA-independent (Supplementary Fig. 5d). Together, these data show that PANDA is an integral part of a BMI1–PRC1 complex and that, both, PANDA and SAFA reciprocally reinforce their affinity for BMI1. By contrast, SAFA binding to EZH2 and other PcG proteins is PANDA- and integrally RNA-independent. Thus, PANDA impacts BMI1–PRC1 rather than PRC2 function.

### PANDA regulates PRC target gene and senescence entry

**Figure 6:**
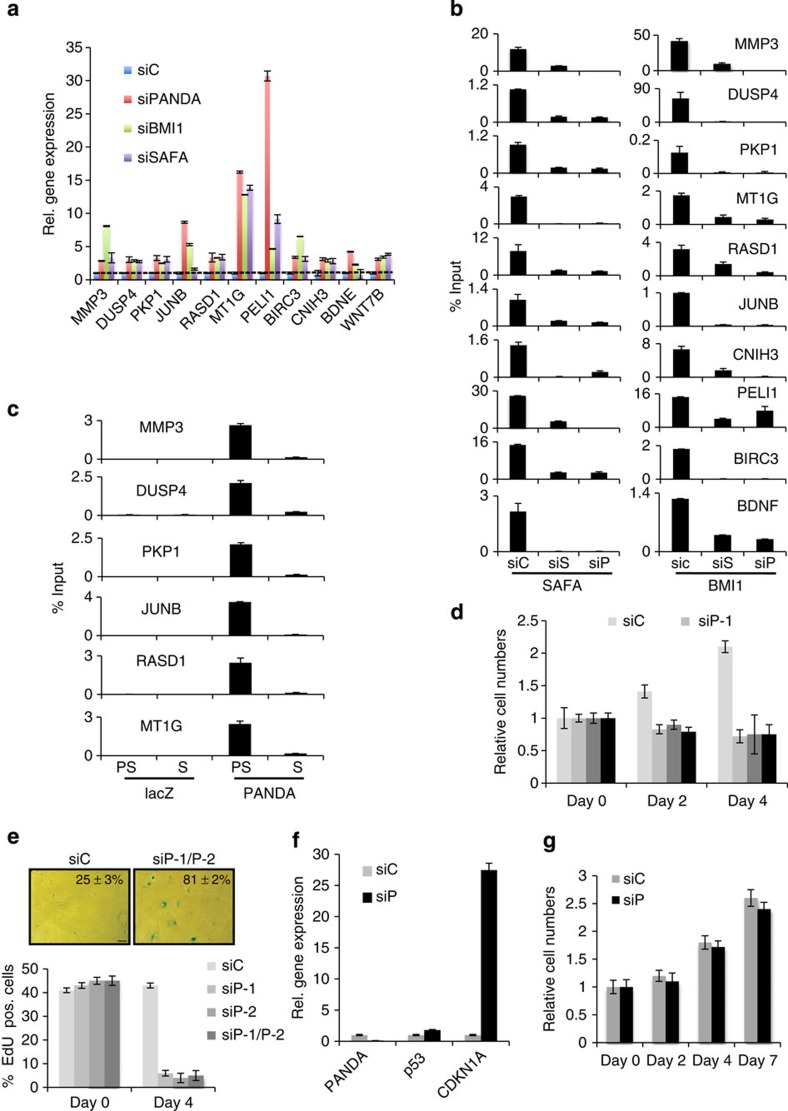
PANDA regulates PRC target gene expression and senescence entry. (**a**) Pre-senescent proliferating BJ fibroblasts were transiently transfected with indicated siRNA duplexes. qRT–PCR on total RNA prepared from respective samples at day 2 of siRNA treatment for indicated genes. Error bars represent the s.d. of three independent experiments; *P*≤0.05, *t*-test. (**b**) SAFA and BMI1 qChIP–PCR of indicated gene promoter regions in siScramble control (siC), siSAFA (siS) and siPANDA (siP)-treated BJ fibroblasts. Error bars represent the s.d. of three independent experiments performed in triplicates; *P*≤0.05, *t*-test. (**c**) PANDA-qChIRP for indicated gene promoters using biotinylated lacZ control and PANDA-specific antisense DNA probes in pre-senescent (PS) and RAS-senescent (S) BJ fibroblasts. Affinity-purified material was analysed by qPCR with gene-specific primers for indicated genes. Error bars represent the s.d. of three independent experiments performed in triplicates; *P*≤0.05. (**d**–**g**) Pre-senescent proliferating BJ fibroblasts were transiently transfected with siScramble control (siC), siPANDA-1 (siP-1), siPANDA-2 (siP-2) or a pool of siPANDA-1 and -2 (siP-1/P-2) for a duration of 4 days. (**d**) Relative (rel.) cumulative cell numbers at day 0, 2 and 4 post siRNA treatment. Error bars represent the s.d. of three independent experiments performed in triplicates; *P*≤0.05, *t*-test. (**e**) Representative micrographs 4 days post siRNA treatment showing BJ fibroblast cell morphology and percentage of SABG-positive cells (upper panel; shown is the result for the siPANDA pool only; scale bar, 20 μm) and bar chart for percentage of Edu positive (pos.) staining cells (lower panel). Error bars represent the s.d. of three independent experiments; *P*≤0.05, *t*-test. (**f**) qRT–PCR on total RNA prepared from respective samples at day 4 of siRNA treatment for p53 and CDKN1A. Shown is the result for the siPANDA pool only. Error bars represent the s.d. of three independent experiments; *P*≤0.05, *t*-test. (**g**) CDKN1A loss rescues siPANDA-induced senescence. CDKN1A knock-out LF1 fibroblasts were transiently transfected either with siScramble control (siC) or siPANDA pool (siP) for a duration of 7 days. Relative cell numbers at day 0, 2, 4 and 7 post siRNA treatment are depicted. Error bars represent the s.d. of three independent experiments; *P*≤0.05, *t*-test.

The aggregate of the above results prompted us to investigate whether or not PANDA regulates SAFA–BMI1–PRC1-mediated transcriptional repression. A recent study by Bracken *et al*.^39^ identified a collection of genes that are de-repressed upon BMI1 depletion. Many of these genes are found de-repressed in senescent cells (Supplementary Data and Supplementary Fig. 6a). We thus examined in siControl, siPANDA, siSAFA and siBMI1-treated proliferating BJ fibroblasts the expression status for a random selection of some of these genes (that is, *BDNF*, *MT1G*, *IL8, BIRC3, JUNB* and *PELI1*), plus for genes that we had already validated as being de-repressed in senescence and by BMI1 depletion (that is, *DUSP4, CNIH3* and *WNT7B*; see Fig. 3b and Supplementary Fig. 3b) and finally for some additional randomly picked PRC target genes that are upregulated in senescent cells (that is, CCNE1, *PKP1, RASD1* and *MMP3*). As summarized in Fig. 6a and Supplementary Fig. 6b, siPANDA (siP), siSAFA (siS) and siBMI1-depletion (siB) led to a strong de-repression by ~3 to 30-fold of *MMP3, DUSP4, PKP1, JUNB, RASD1, MT1G, PELI1, BIRC3, CNIH3*, *BDNF*, *WNT7B, IL8* and *CCNE1* compared with siControl (siC)-treated cells. Moreover, using qChIP–PCR, we could demonstrate that de-repression of PRC-responsive genes in PANDA- and SAFA-depleted cells led to a strong reduction in the physical presence of BMI1 (Fig. 6b) and H2AK119_ub1_ (Supplementary Fig. 6c, compare lanes 7, 8 and 9) at respective target genes, which is comparable with what is observed in siBMI1-treated cells (Supplementary Fig. 6d, compare lanes 9 and 10). Interestingly, PANDA silencing also induced a reduction of SAFA binding at interrogated promoter targets. This implies that, although SAFA has the potential to specifically and autonomously bind non-chromatized ‘naked’ DNA as used in the fully recombinant electrophoretic mobility shift assay (see Fig. 4c), in a native chromatin environment, PANDA seems to impact the dynamics (either on or off rate) of SAFA binding and thus the effective residence time at any given gene target. The global level of H2AK119_ub1_ was not altered in siPANDA- (siP) compared with siControl (siC)-treated cells indicating that depletion of PANDA akin to SAFA (Supplementary Fig. 3f) only leads to gene-specific and global changes in H2AK119_ub1_ (Supplementary Fig. 6e). The above observations led us to examine if PANDA acts directly at PRC targets by employing chromatin-isolation by RNA purification (ChIRP)^47^ followed by qRT–PCR (qCHIRP) (Fig. 6c). To this end, we designed a set of 10 unique complementary oligonucleotides to PANDA RNA and as a negative control five complementary probes that target bacterial lacZ mRNA. In concordance with the notion that PANDA acts as a co-repressor at the same sites as PRCs in pre-senescent proliferating cells (PS), we could specifically pull down PANDA RNA and enrich for a selection of PRC target promoters with PANDA, but not lacZ probes, in these cells, whereas this failed in senescent (S) cells although we efficiently enriched for PANDA in these cells as well (Supplementary Fig. 6f). On the basis of the above data, we surmised that PANDA depletion could impact the proliferative potential of pre-senescent fibroblasts by destabilizing BMI1–PRC1, thus, leading to de-repression of pro-senescence PRC targets and an ensuing onset of senescence. Indeed, extended siRNA-mediated PANDA depletion induced a proliferative arrest in pre-senescent BJ fibroblasts within 4 days (Fig. 6d) with canonical features of senescence including increased SABG activity and decreased incorporation of 5-ethynyl-2 deoxyuridine (EdU) into DNA (Fig. 6e), modest to strong upregulation of *p53* and pro-senescence PRC targets *CDKN1A* (Fig. 6f), *DUSP4*, *JUNB* (see Fig. 6a), *IL8* and *CCNE1 (*Supplementary Fig. 6b) and downregulation of cell cycle genes *CCNE2*, *CCNA2*, *CDK1*, *CDK2*, *MCM3*, *PCNA*, as well as of *LB1* (Supplementary Fig. 6g). Importantly, siPANDA-induced senescence neither occured in CDKN1A knock-out fibroblasts^48^ (Fig. 6g) nor in PANDA and CDKN1A co-silenced cells indicating that CDKN1A constitutes a crucial downstream target for PANDA-dependent senescence (Supplementary Fig. 6h,i). Collectively, our results indicate that PANDA, in conjunction with SAFA, is a transcriptional co-repressor of PRC target genes through recruitment of BMI1–PRC1 to respective promoters and an ensuing H2AK119 ubiquitination of the latter in pre-senescent proliferating cells. Moreover, our data show that PANDA levels are instrumental for cell-fate decisions since PANDA depletion in proliferating cells led to a senescent proliferative arrest by decomposition of the SAFA–PANDA–BMI1 complex and subsequent de-repression of the CDKN1A growth-inhibitory gene.

### PANDA regulates NF-YA function and senescence exit

**Figure 7:**
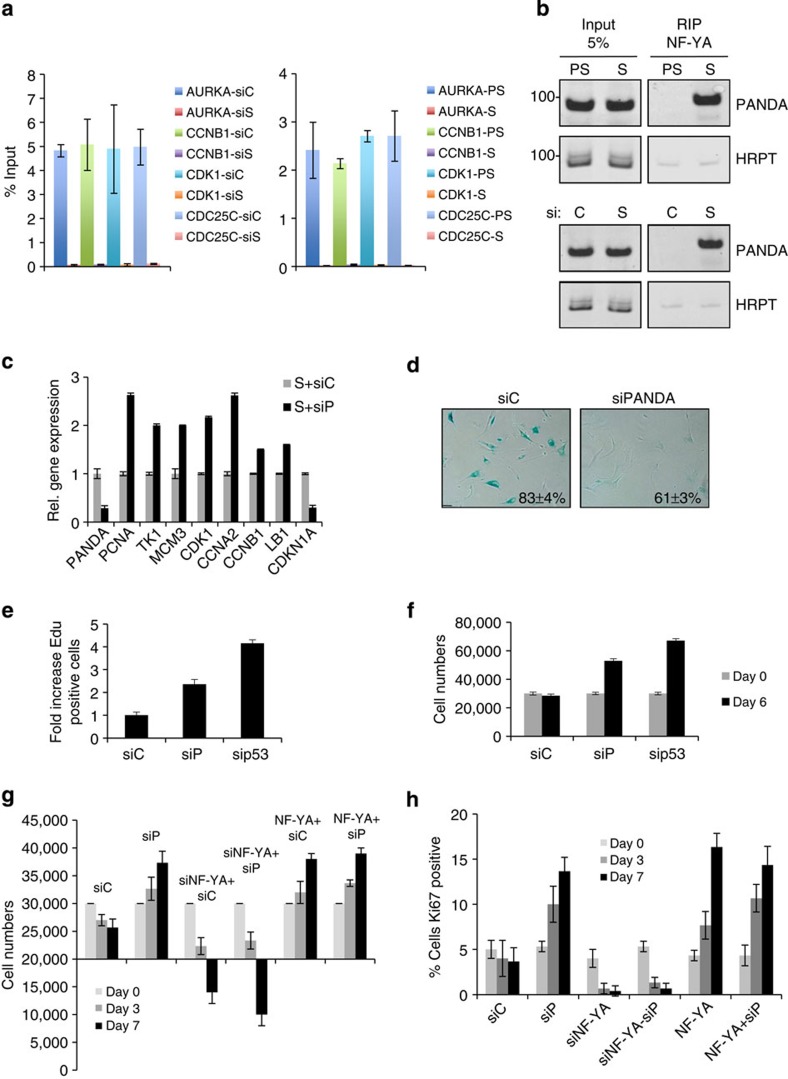
PANDA regulates NF-Y/E2F-coregulated expression of pro-proliferation genes and senescence exit. (**a**) NF-YA qChIP–PCR for indicated gene promoters in siScramble control (siC) and siSAFA (siS) treated pre-senescent proliferating BJ fibroblasts 3 days post treament (left panel) and in pre-senescent proliferating (PS) and RAS-senescent (S) BJ fibroblasts (right panel). Error bars represent the s.d. of three independent experiments performed in triplicates; *P*≤0.05, *t*-test. (**b**) Representative (*n*=3) native RIP in lysates from pre-senescent proliferating (PS) and RAS-senescent (S) BJ fibroblasts (upper panel) and siScramble control (siC) or siSAFA (siS)-treated presenesent proliferating BJ fibroblasts (lower panel) using antibody to NF-YA. Precipitated material was analysed for PANDA and HRPT as negative control by RT–PCR. (**c**) Relative gene expression of indicated genes as measured by qRT–PCR on total RNA prepared from fully senescent RAS BJ fibroblasts (S) transfected either with siScramble control (siC) or siPANDA pool (siP) for a period of 48 h. Error bars represent the s.d. of three independent experiments performed in triplicates; *P*≤0.05, *t*-test. (**d**) Representative (*n*=3) micrographs of siC and siPANDA expressing BJ fibroblasts showing their cell morphology, SABG staining and percentage of SABG-positive cells (scale bar, 20 μm). (**e**) Relative fold increase in Edu incorporation of same cell populations including also sip53-treatment serving as positive control for senescence exit; 200 cells per count; *P*≤0.05, *t*-test. (**f**) Cell numbers of same cell populations 6 days post treatment. Error bars represent the s.d. of three independent experiments; *P*≤0.05, *t*-test. (**g**,**h**) NF-YA is instrumental for senescence survival and escape. RAS-senescent cells depleted for (siNF-YA) or transiently overexpressing NF-YA (pSG5-NF-YA) in the presence or absence of siPANDA (siP) and scored for (**g**) cell numbers and (**h**) percentage of cells staining positive for Ki67 at 0, 3 and 7 days after treatment. Experiment was performed three times *n*=3; *P*≤0.05, *t*-test.

PANDA levels increased in senescent cells (see Fig. 4a), whereas SAFA decreased (Fig. 1b), and the interaction between PANDA and residual SAFA was reduced in senescent cells (see Fig. 5b). The questions that naturally arise is: where does PANDA go in senescent cells and how may PANDA aid in the implementation of senescence? Recently, PANDA was shown to function as a molecular decoy for the transcription factor NF-YA to inhibit activation of an apoptotic gene expression programme. Aside from controlling a number of pro-apoptotic genes, NF-YA also co-regulates, in unison with E2Fs, a bevy of genes involved in cell cycle progression^3^, many of which are permanently repressed in senescent and SAFA-depleted cells (see Supplementary Fig. 1). We thus considered that PANDA could sequester NF-YA from occupying target genes, thus, impeding the expression of NF-YA:E2F-coregulated pro-proliferation targets. To this effect, we first determined the physical presence of NF-YA on selected, cognate E2F targets in pre-senescent (PS), senescent (S), SAFA (siS)-depleted and siControl (siC) cells by qChIP–PCR. Consistent with the idea that PANDA sequesters NF-YA from its targets we found that in senescent (Fig. 7a, right panel) and SAFA-depleted cells (siS) (Fig. 7a, left panel) NF-YA binding to its gene targets including *AURKA*, *CCNB1*, *CDK1* and *CDC25C* was severely compromised when compared with respective controls although expression of NF-YA remained unchanged in senescent and siSAFA-treated cells when compared with control cells (Supplementary Fig. 7a). Importantly, NF-YA presence at promoters could be rescued by overexpression of SAFA in siSAFA-treated cells demonstrating the specificity of the siSAFA treatment (Supplementary Fig. 7b, compare lanes 8 and 9). To examine whether or not NF-YA would differentially interact with PANDA in senescent (S) versus pre-senescent proliferating (PS) and siSAFA (siS)- versus siControl (siC)-treated BJ fibroblasts, we performed endogenous RIP analysis using an antibody to NF-YA. As summarized in Fig. 7b and Supplementary Fig. 7c, we found NF-YA to bind to PANDA exclusively in senescent (upper right panel) and SAFA-depleted cells (lower right panel), whereas in the respective control cells, the interaction was barely detectable, the latter also illustrating the specificity of the interaction. There was also no enrichment for abundant HRPT mRNA in the NF-YA pull-downs further confirming the specificity of the PANDA:NF-YA interaction. The exclusive interaction between PANDA and NF-YA in senescent cells was exactly reciprocal to what we had observed for the association between PANDA and SAFA in proliferating cells (see Fig. 5b). These data thus show that PANDA switches between NF-YA and SAFA complexes in a cell-fate dependent manner. On the basis of these results we inferred that acute PANDA depletion from fully established senescent cells might restore NF-YA function, thus enabling re-activation of a pro-proliferative target gene expression programme and ultimately restoring cell proliferation. Indeed, siRNA-mediated PANDA depletion over a period of 6 days in fully senescent BJ fibroblasts (S+siP) caused a transient upregulation of pro-proliferation NF-YA:E2F coregulated targets such as PCNA, TK1, MCM3, CDK1, CCNA2, CCNB1 and LB1 and a strong downregulation of pro-senescence PRC target CDKN1A when compared with siControl-treated senescent cells (S+siC) (Fig. 7c). The activating effect of NF-YA on the expression of these genes was further supported by the downregulation of their expression in NF-YA-depleted cells (Supplementary Fig. 7d). Interestingly, the PANDA and SAFA levels obtained after siRNA-mediated PANDA silencing were comparable to those of siC-treated pre-senescent proliferating cells (PS+siC) (Supplementary Fig. 7e), which may favour the repartioning of PANDA from NF-YA back to SAFA to activate PRC1 function leading to re-repression of pro-senescence PRC targets as exemplified here for *CDKN1A*. Consistent with the creation of an anti-senescence, pro-proliferative intracellular environment, we observed an ~1.5-fold reduction in the number of SABG-positive cells (Fig. 7d), a ~2-fold increase in actively replicating cells as evidenced by an incorporation of 5-ethynyl-2 deoxyuridine (EdU) into DNA (Fig. 7e) as well as a ~2-fold increase in cell numbers in PANDA-depleted compared with siC-treated senescent cells, which is comparable to senescent cells treated with sip53 serving as a positive control for senescence escape^49^ (Fig. 7f). To corroborate further the importance of NF-YA for senescence exit, we transiently transfected fully RAS-senescent cells with siNF-YA or an NF-YA expression construct (pSG5-NF-YA) either alone or together with siPANDA (siNF-YA/siP and NF-YA/siP). As shown in Fig. 7g,h and Supplementary Fig. 7f, overexpression of NF-YA led to senescence escape while its silencing caused cell death of senescent cells as indicated by a dramatic loss in cell numbers and cytomorphological changes. Thus, NF-YA is an instrumental factor both for the escape from senescence as well as survival of senescent cells underlining the importance of PANDA as a molecular decoy for the transcription factor NF-YA. Of note, we detected a significant negative correlation between PANDA, CDKN1A and SAFA, BMI1, EZH2 transcript levels in human hepatocellular carcinomas (HCC) when compared with normal liver samples with PANDA and CDKN1A levels significantly reduced (~2-fold) and SAFA (~2-fold), BMI1 (~1.7-fold) and EZH2 (~4.5-fold) levels significantly increased. Importantly, PANDA and CDKN1A downregulation was independent of P53 mutational status in HCC indicating that there is positive selective pressure for low PANDA and CDKN1A and elevated SAFA, BMI1 and EZH2 expression levels. This implies that repressive forces like SAFA–PRC complexes rather than the lack of active forces like p53 may control PANDA and CDKN1A expression in these tumours, which is in line with published results for EZH2-dependent repression of CDKN1A in melanomas^33^ (Supplementary Fig. 7f and Supplementary Table 1). Collectively, our results demonstrate that PANDA has a critical role for the establishment and maintenance of the senescence phenotype by facilitating the repression of a pro-proliferative gene expression programme driven by NF-YA and E2F transcription factors, at least in part, by working as a decoy molecule for NF-YA. Accordingly, a decrease in PANDA levels in fully senescent cells creates a cellular milieu conducive for proliferation including the re-activation of NF-YA:E2F-function and the re-repression of pro-senescence PRC-responsive targets like *CDKN1A*.

## Discussion

**Figure 8:**
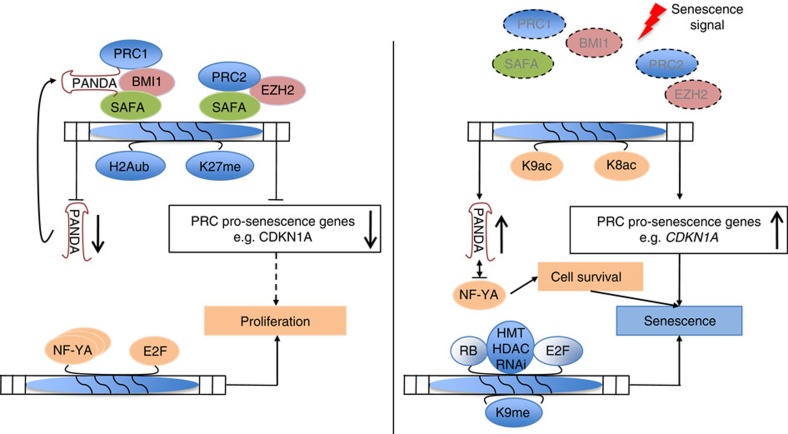
Model for SAFA–PANDA–PRC-mediated coordination of proliferation and senescence. In proliferating cells (left panel of cartoon) SAFA and PANDA recruit PRC2 and BMI1–PRC1 complexes to generate repressive histone marks H3K27_me3_ and H2AK119_Ub1_ and dampen transcription of pro-senescence PRC target genes such as coregulated targets *CDKN1A* and noncoding transcript *PANDA*. PANDA acts in an autoregulatory loop with SAFA–BMI1–PRC1 to regulate its own trancription as well as function of the complex. The staggered arrow indicates that low stoichiometric concentrations of certain pro-senescence factors may be beneficial for proliferation as is the case for CDKN1A, which operates as an assembly factor for active cyclin D–Cdk4/6 complexes^60^. NF-YA affinity for PANDA is low and it thus is free to induce-coactivate with E2F transcription factors, the expression of proliferation-promoting genes. In cells undergoing senescence (right panel of cartoon), SAFA–PRC2 and SAFA–PANDA–BMI1–PRC1 complexes disintegrate and expression of pro-senescence genes including PANDA and CDKN1A is upregulated (depicted here by H4K8- (K8ac) and H3K9-acetylation (K9ac)). PANDA now blocks proliferation by decoying NF-YA. NF-YA is also required for survival of senescent cells. CDKN1A mediates cell cycle arrest by activating tumour suppressor protein Rb, which assembles E2Fs into co-repressor complexes including histone methyl transferases (HMT), histone deacytylases (HDAC) and components of the RNAi machinery to repress expression of proliferation-promoting genes (depicted here by H3K9-trimethylation, K9me) and thus to enforce senescence arrest.

Our knowledge about the gene-regulatory mechanisms that govern the onset and maintenance of the senescence phenotype must still be considered fragmentary. We have recently shown that microRNAs (miR) and miR-binding protein argonaute 2 (AGO2) are critical for senescence induction by facilitating transcriptional gene silencing of E2F-regulated proliferation-promoting genes during senescence^5^. We now extend the crucial function of noncoding RNAs (ncRNAs) and RNA-binding proteins (RBPs) in senescence-associated gene regulation to another class of ncRNAs and RBPs. In the present study, we provide compelling evidence for a critical and novel function of scaffold-attachment-factor A (SAFA alias hnRNPU) and lncRNA PANDA in the regulation of proliferation and cellular senescence (Fig. 8). One major finding is that SAFA and lncRNA PANDA physically and functionally interact to repress senescence-promoting/enforcing genes in proliferating cells by effectively recruiting BMI1–PRC1 and EZH2–PRC2 to promoters of these genes. This is consistent with the role SAFA has in the deposition of the PRC2-catalysed K27_me3_ negative chromatin mark in X-inactivation^23^. In senescent cells, SAFA–PANDA–PRC interactions are disrupted and pro-senescence genes are de-repressed and congruent with this, acute SAFA and PANDA depletion in pre-senescent proliferating cells equally leads to de-repression of these genes and induces a senescence arrest. On the basis of these data, we speculate that in many cases the binding between PRC complexes and lincRNAs and their recruitment to target genes may not be direct but is likely mediated by multimodular DNA/RNA-binding proteins such as SAFA/hnRNPU or the recently identified hnRNPK^45^. Due to their multimodular nature, these proteins naturally lend themselves to multilateral crosstalk and they are thus able to integrate inputs from different signal transduction pathways and to control outputs. In conclusion, SAFA–PANDA-dependent PRC recruitment and repression of respective response genes entails a combination of self-reinforcing DNA–protein, RNA-protein and protein–protein interactions.

Another major finding of our study is that PANDA changes partners depending on cell fate. PANDA and SAFA predominantly interact in pre-senescent, proliferating cells although PANDA expression is strongly increased in senescent cells. In the latter, PANDA is primarily bound by NF-YA. The reduced association between SAFA and PANDA can be explained, at least in part, by the decrease of SAFA protein levels in senescent cells. Yet, we believe that the changes in affinity are not solely the result of reduced SAFA levels but most likely are also regulated by posttranslational modifications of the two proteins. Indeed, SAFA and NF-YA are subject to different types of posttranslational modifications including phophorylation, acetylation and ubiquitination^50^,^51^. In senescent cells, we find that PANDA inhibits the expression of crucial E2F-regulated proliferation-promoting genes by sequestering the transcription factor NF-YA from occupying E2F/NF-YA target gene promoters. PANDA depletion here leads to re-activation of these genes and to senescence exit. This finding underscores the important function of NF-YA in fostering a cooperative interaction with E2F transcription factors to regulate cell cycle genes^3^,^4^ and also propounds the notion that the ability of E2Fs to functionally interact with a specific promoter element is dictated by the presence of other partner proteins or RNAs as we have previously described for miR-AGO2-mediated transcriptional silencing of E2F target genes^4^,^5^. Remarkably, we also find that depletion of NF-YA from fully senescent cells leads to cell death demonstrating a function of NF-YA beyond its regulation of E2F targets in the survival of senescent cells. This is remarkable because senescent cells are inherently resistant to undergo apoptosis^52^,^53^. A function for NF-YA in cell death is well documented in mammalian cells, however, only in the context of proliferating cells where NF-YA depletion or overexpression was shown to drive cells towards p53 or E2F1-mediated apoptosis^26^,^54^. Thus, our data opens a new line of investigation towards a specific role for NF-YA in survival of senescent cells and this may be of future therapeutical utility to specifically eliminate senescent cells as an accumulation of these cell can have detrimental organismal effects in the long run^55^.

From the above it follows that PANDA may be viewed as a ‘locking device’ that confines cells in their current proliferative state. Accordingly, in proliferating cells, PANDA depletion causes senescence entry whereas in fully senescent cells PANDA depletion leads to senescence exit. The latter may be exploited by tumours because we observed a significant downregulation of PANDA in HCCs, which was correlated with increased expression levels of SAFA, BMI1 and EZH2, all of which are repressors of PANDA expression.

In summary, we demonstrate that SAFA and PANDA have a crucial role for senescence entry and exit and thus extends the role that PANDA has in cell-fate decisions beyond apoptosis^19^. While we leave open the possibility that SAFA and PANDA may regulate proliferation and senescence through additional mechanisms, we provide a series of evidence suggesting that modulation of PRC and NF-YA function(s) has a major role in these processes. In addition, our data throw a new light on the role of PcG proteins in fully differentiated cells: their main function here seems to assist in dynamically modulating the expression of genes involved in signalling and proliferation rather than to just maintain epigenetic memory of their silencing.

## Methods

### Vectors and viruses

Retroviral vector pBABE-RAS^V12^ and pLNCX^Neo^-ER:Ras^V12^ was as reported^5^,^24^. To activate ER:Ras^V12^, infected cells were treated with 100 nM 4OHT or EtOH as control. Lentiviral shSAFA constructs TRCN0000001297, TRCN0000001298 and shControl SHC002 were from SIGMA-Mission. Lentiviral infection was performed by standard procedures. Full-length PANDA was gene synthesized, sequence-verified (MWG Eurofin) and cloned into pcDNA3.1. SAFA full-length cDNA was cloned into pGEX-4T vector. Flag-SAFAwt, Flag-SAFAΔSAF, Flag-SAFAΔSPRY, Flag-SAFAΔRGG vectors were a kind gift of Dr S. Nakagawa. HA-Bmi1 vector was kind gift of Dr M. vanLohuizen. Flag-SUZ12 and Flag-EZH2 vectors were a kind gift of Dr D. Reinberg. pcDNA-FLAG-NF-YA was a kind gift of Dr R. Mantovani.

### Cell culture

Primary human diploid fibroblast strains, BJ (neonatal foreskin) and WI38 (fetal lung) were purchased from ATCC. LF1 p21 knock-out fibroblasts were as described in Brown *et al*.^48^ and provided by U. Herbig. Culturing of cells and infection of primary human diploid fibroblasts by retroviral-mediated gene transfer were performed as previously described^5^. Briefly, Phoenix packaging cells were transfected by calcium phosphate with the respective retroviral constructs. Forty-eight hours posttransfection supernatants were recovered and filtered and target cells were infected for a total of three times. Pharmacological selection was started 24 h post infection. WI38 primary human lung fibroblasts were grown in DMEM supplemented with 10% fetal bovine serum (FBS) and penicillin/streptomycin. All cells were maintained at 37 °C under an atmosphere containing 3% (for primary fibroblasts) or 21% (for other cell lines) O_2_. RS cells were generated by proliferative exhaustion and were used for experiments when cell cultures went through ≤1 population doubling per 2 weeks, were ≥80% positive for SABG staining and Ki67 negative.

### Antibodies

All antibodies were used at a dilution of 1:1,000. Actin (clone AC-74), SAFA/hnRNPU (sc32315), NF-YA (sc-10779), normal rabbit (sc-2027) and mouse IgG (sc-2025), HA (sc-57592), H3K9-trimethyl (#9754), H3K4-trimethyl (#9751), H3K27-trimethyl (#9733), H3K36-trimethyl (#4909), H3K79-trimethyl (#4260), H3K56-acetyl (#4243), H3K9-acetyl (#9649), H4K5-acetyl (#9672), H3K18-acetyl (#9675), H3K27-acetyl (#4325), SUZ12 (#3737), Ezh2 (#5246), Ring1A (#2820), RING1B (#5694), Bmi1 (#6964), H2AubK119 (#8284), phospho-specific Rb (#9969) from Cell Signalling; H3K27-trimethyl (#07-449), H3K4-acetyl (#07-328), H3K9-acetyl (#07-352) all from Millipore, H3K27-acetyl (ab4729), H4K8-acetyl (ab15823), Anti-Histone H2A (ab18975) from Abcam; LB1 (Ab-1, Calbiochem), beta-Tubulin (CP-06, Calbiochem), rabbit polyclonal Ki67 (Vectorlabs), CDKN1A (sc-397 and -469), CDKN1A (OP-76, OP-68, Calbiochem), Flag (Sigma, F3165), HP1α (2HP- 2G9-AS, Euromedex). All fluorochrome-tagged secondary antibodies were from Molecular Probes.

### Senescence analysis

Senescence was assessed using several assays as previously published^5^,^24^. Briefly, proliferative capacity was determined by life-span studies, immunoblot analyzes of senescence biomarker proteins, indirect immunofluorescence using Ki67-specific antibody staining (Boehringer) or Edu incorporation using Click-iT (Invitrogen) as per the manufacturer’s instructions. Cells were also co-stained for SABG activity.

### Co-immunoprecipitation and western blotting

Cells were extracted in 300 mM NaCl, 10 mM Tris-pH 8.0, 0.5% NP-40, 1 mM EDTA and protease inhibitors. For immunoprecipitations, equal amounts of lysate were incubated with 1–5 μg of respective antibodies over night at 4 °C. Precipitates were purified and analysed by western blotting by standard procedures using indicated antibodies at a dilution of 1:1,000. When immunoprecipitation was not performed, western blotting of total protein lysates was peformed according to classical procedures. Quantification was analysed densitometrically using ImageJ64. Uncropped blots or gels are provided in Supplementary Fig. 8.

### Chromatin immunoprecipitation and histone association assay

ChIP was performed using 1–2 μg of respective antibodies as per the manufacturer’s instructions (#9003S, Cell Signalling) and as previously described^5^. All ChIP primers against PRC targets were designed according to published BMI1, EZH2 and H3K27_me3_ promoter profiles available on the UCSC Genome Browser^56^,^57^ (Supplementary Table 2). When histone association assay was performed, crosslinked cellular lysates were immunoprecipitated with the respective histone antibodies as described. Co-immunoprecipitates were analysed by western blot using indicated antibodies.

### Immunofluorescence and Proximity-Ligation Assay

Cells were prepared and immunolabelled with primary (1:200 to 1:600 dilution) and secondary antibodies (1:800 dilution) as previously described. DUOLINK Proximity-Ligation Assay (PLA) was performed according to vendor’s instructions (SIGMA). Fluorescence images were acquired on a Zeiss Axiovert 200 (Leica, Germany) using a × 63 numerical aperture (NA)=1.46 oil objective for high magnification images and a × 20 NA=0.16 objective for low magnification images. High-resolution images were acquired as a z-stack with an ~0.2 μm z-interval. When necessary, sequential scanning was used to prevent crosstalk between channels for samples with multiple fluorophores. PLA signals were quantified of at least 100 nuclei. High-resolution (× 63 NA=1.46) images from single scans were analysed in Fiji (NIH) to calculate the density of PLA puncta. Images were first smoothed, and a threshold was selected manually to discriminate PLA puncta from background fluorescence. Once selected, this threshold was applied uniformly to all the images in the sample set. The built-in macro ‘Analyze Particles’ was then used to count and characterize all the objects in the thresholded image. The remaining objects were counted as PLA puncta.

### Transfection and siRNA treatment

Cells were transfected with indicated vector constructs using X-tremeGENE HP DNA transfection reagent (Roche) or siIMPORTER (Millipore). For siRNA transfection experiments, cells were transfected at 100 nM. All siRNA duplexes were Smart Pools (Dharmacon) except for SAFA siRNAs, which were from Qiagen (S102780540 and S102781002)(Supplementary Table 3) and have been previously validated. Respective scrambled sequences were used as negative control. Cells were collected at indicated time points and analysed.

### qRT–PCR analysis of mRNA

Total RNA and cDNA preparation were performed by standard procedures. Real-time PCR (qPCR) was conducted using Qiagen QuantiTec primers or gene-specific primers (Supplementary Table 3) for indicated genes with Roche LightCycler and Roche Absolute QPCR SYBR Green Capillary Mixes. In all assays cyclophylin (CYP), HRPT, actin and GAPDH served as normalization controls.

### Native RNA immunoprecipitation

Cells were lysed in NP-40 lysis buffer (50 mM Tris HCl, ph 7.4, 150 mM NaCl, 1% NP-40 and Protease inhibitor cocktail) in the presence or absence of RNAse A (100 μg ml^−1^) and cleared lysates were immunoprecipitated with indicated antibodies. Immune complexes were purified with Protein-A/G-coupled dynabeads (Life Technologies) and subjected to RNA isolation by Nucleospin RNA II purification kit (Clonetech). RNA was reverse transcribed by SuperScript III reverse Transcriptase (Life Technologies). cDNA was used as a template in PCR amplifications with gene-specific primers.

### Electrophoretic mobility shift assay

Electrophoretic mobility shift assay (EMSA) was performed as described previously^58^. Briefly, full-length GST-SAFA and GST proteins were expressed in BL21 cells and purified using Glutathione Sepharose (GE) affinity chromatography. The bacterial cell pellet was lysed in five volumes of lysis buffer (200 mM Nacl, 20 mM Tris HCl pH 7.4, 1 mM EDTA, 0.5 mM PMSF, one tablet of protease inhibitor cocktail (Roche) per 10 ml of lysis buffer). Cleared lysate was passed through a pre-equilibrated Glutathione Sepharose resin column. GST and GST-SAFA proteins were eluted from the column using reduced glutathione-containing elution buffer (25 mM glutathione, 50 mM Tris-pH 8.8, 200 mM NaCl). Both the proteins were dialyzed against electrophoretic mobility shift assay binding buffer. Dialyzed proteins were aliquoted and flash frozen in liquid nitrogen and stored at −80 °C until use. Promoter regions of CDKN1A were amplified by PCR and end labelled with ^32^P and PNK enzyme as per the instruction of the manufacturer. Radiolabeled fragments were gel purified followed by phenol:chloroform extraction. Binding reactions were performed in a 20 μl total volume containing 10 mM HEPES (pH 7.9), 1 mM dithiothreitol, 50 mM KCl, 2.5 mM MgCl_2_, 10% glycerol, 0.5 ig salmon sperm DNA and 1 to 500 mg of recombinant protein. Samples were preincubated at 4 °C for 5 min before the addition of probe. After 15 min of incubation at 4 °C temperature, the bound products were resolved by 8% native polyacrylamide gel electrophoresis. The gels were dried under vacuum and exposed to X-ray film.

### Chromatin-Isolation by RNA Purification

Chromatin-Isolation by RNA Purification (ChIRP) was performed as described^47^ with minor modifications. MNase treatment was used instead of sonication. PANDA and LacZ probes were designed by online programme at ‘singlemoleculefish.com’ (Supplementary Table 4). Antisense DNA probes were synthesized (MWG Eurofin) and biotinylated with Pierce 3′ End desthiobiotinylation Kit (#20163) according to manufacturer’s instructions.

### Human tumour samples

All the samples were part of a previously published retrospective cohort of HCCs^59^. The recruitment of patients respected the Declaration of Helsinki. The research programme received approval from an Institutional Review Board (RBM no. 2005-019). The original authors gave their consent for the samples to be used.

## Author contributions

O.B. and P.K.P. conceived the project. O.B., P.K.P. and R.D.D. carried out the experiments. O.B and P.K.P. analysed the data. A.M./P.P. carried out the primary tumour analysis. O.B. wrote the manuscript and supervised the work. A.D. and A.M. provided valuable support.

## Additional information

**How to cite this article:** Puvvula, P. K. *et al*. Long noncoding RNA PANDA and scaffold-attachment-factor SAFA control senescence entry and exit. *Nat. Commun.* 5:5323 doi: 10.1038/ncomms6323 (2014).

## Supplementary Material

Supplementary Figures and Supplementary Tables Supplementary Figures 1-8 and Supplementary Tables 1-4

Supplementary Data 1 List of PRC targets up-regulated in cells undergoing oncogenic RAS-induced senescence
