# Supplementary material for: Long noncoding RNA PANDA and scaffold-attachment-factor SAFA control senescence entry and exit
Source: Nat Commun. 2014 Nov 19;5:5323. doi: 10.1038/ncomms6323 (PMC4263151; doi:10.1038/ncomms6323)
Supplement: Supplementary Figures and Supplementary Tables — Supplementary Figures 1-8 and Supplementary Tables 1-4 [file ncomms6323-s1.pdf]

Supplementary Figure 1

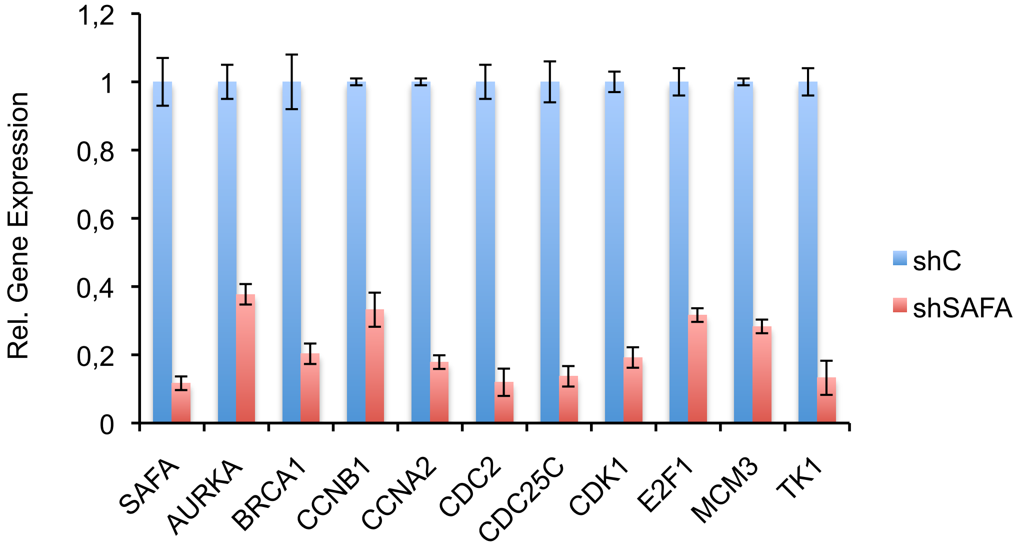

Supplementary Figure 1. SAFA repression actively contributes to senescence entry. Presenescent proliferating BJ fibroblasts were transduced either with pLKO.1-shC or pLKO.1-shSAFA-1. qRT-PCR on total RNA prepared from respective samples at day 6 post drug selection for indicated E2F-regulated pro-proliferation target genes. Error bars represent the s.d. of three independent experiments; P ≤ 0,05, *t*-test.

Supplementary Figure 2

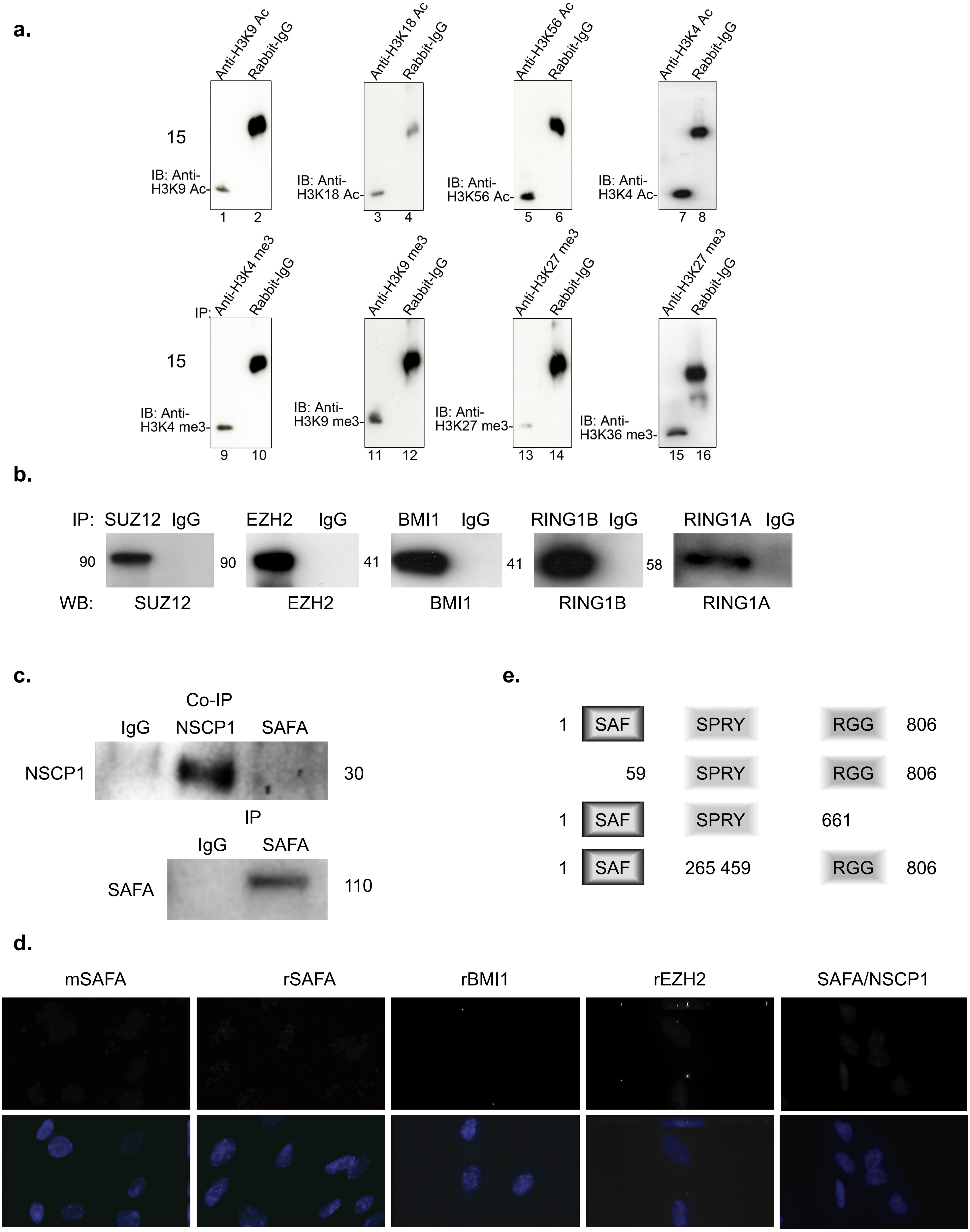

Supplementary Figure 2. SAFA repression actively contributes to senescence entry. a. Efficacy of antibodies against histone modifications used in histone-association-assay of Figure 2A. **b.** Efficacy of antibodies to SUZ12, EZH2, BMI1, RING1A and RING1B antibodies used in co-immunoprecipitation experiments of Figure 2C. **c.** Representative (n = 3) co-immunoprecipitation (Co-IP) of endogenous SAFA with PcG protein NSCP1 in proliferating BJ fibroblasts using antibodies to NSCP1 and SAFA or IgG. Immunoprecipitates were Western-blotted with antibodies to NSCP1 and SAFA. **d.** Specificity of proximity-ligation assay (PLA) was tested by omitting one primary antibody out of the two used in Figure 2D. Shown are negative technical controls for mouse (m) anti-SAFA- and rabbit (r) anti-SAFA-, BMI1- and EZH2-specific antibodies. Also shown is a SAFA-NCSP1 negative biological control. DAPI was used to counterstain nuclear DNA. Scale bar 20 μm. **e.** Schematic drawing of FLAG-tagged SAFA mutants used in Figures 2E and -5C.

Supplementary Figure 3

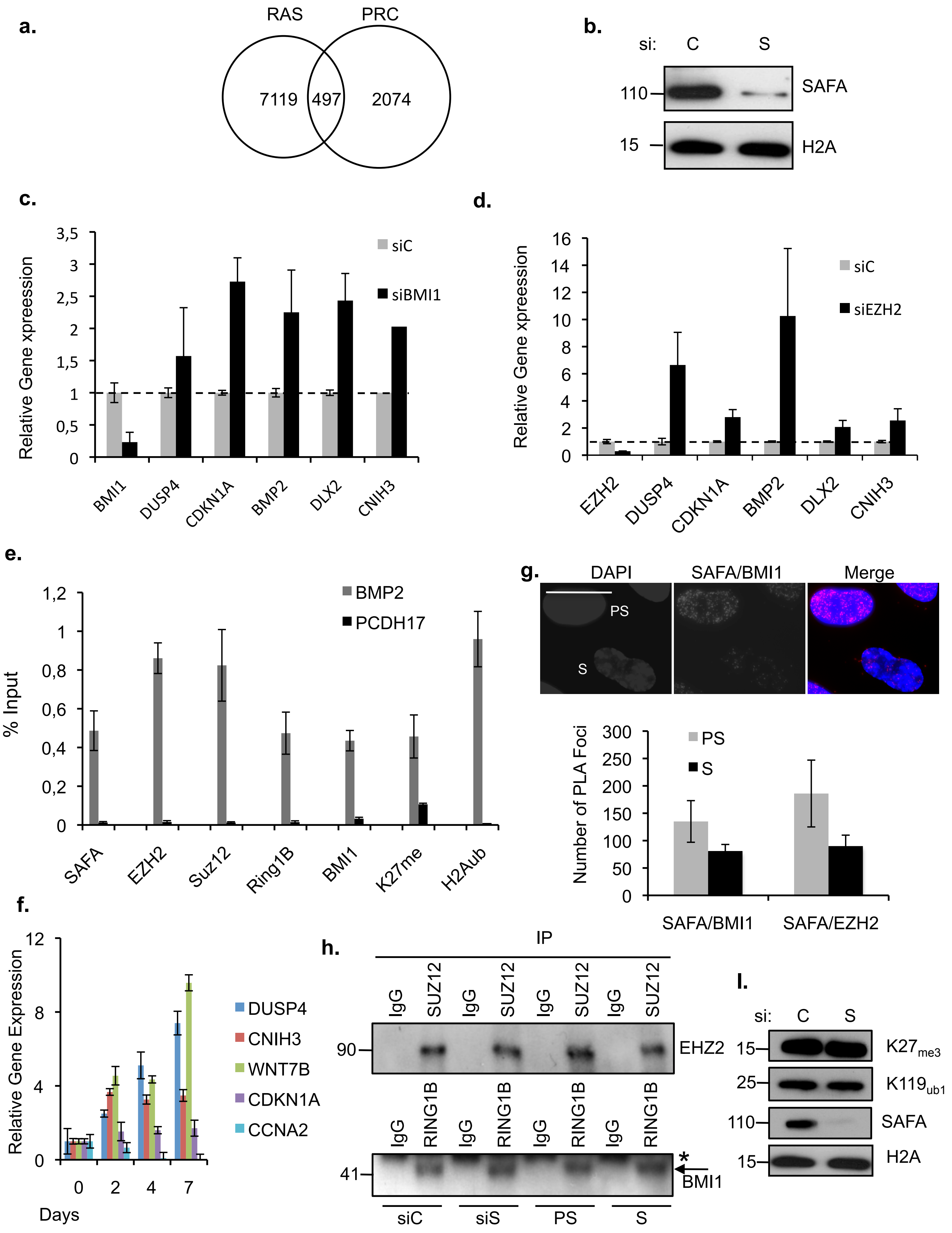

Supplementary Figure 3. SAFA regulates expression of pro-senescence genes. a. Venn diagram depicting number of potential PRC targets derepressed by at least 2-fold in RAS senescence. **b.** Silencing efficiency of siRNA pool against SAFA used in this study as determined by western Blot. **c-d.** Presenescent proliferating BJ fibroblasts were transiently transfected with siScramble control (siC), **c.** siBMI1 or **d.** siEZH2. qRT-PCR on total RNA prepared from respective samples at day 2 of siRNA treatment for indicated genes. Error bars represent the s.d. of three independent experiments; $P \leq 0{,}05$, *t*-test. **e.** SAFA, EZH2, SUZ12, RING1B, BMI1, $H3K27_{me3}$ ($K27_{me}$) and $H2A_{Ub1}$ ($H2AK119_{Ub1}$) qChIP-PCR for BMP2 (positive control) and PCDH17 (negative control) was performed in proliferating BJ fibroblasts. **f.** Relative gene expression of indicated genes as measured by qRT-PCR at the indicated time-points on total RNA prepared from EtOH- and 4OHT-treated ER:Ras^{V12} BJ fibroblasts. Note the gradual increase of SAFA-PRC-regulated genes and decrease of proliferation-promoting genes (e.g. CCNA2) starting as soon as day 2 post-tamoxifen treatment. **g.** Representative proximity-ligation assay (PLA) for SAFA and PcG proteins BMI1 and EZH2 in proliferating and RAS senescent (S) BJ fibroblasts. For clarity PS and S cells were mixed equally. Note the characteristic SAHF formation in RAS cells. Each dot represents a positive protein-protein interaction in PLA. Also shown is the quantification of PLA interactions. DAPI was used to counterstain nuclear DNA in merged images. Scale bar 20 μM. Error bars represent the s.d. of three independent experiments; $P \leq 0{,}05$ ($n = 100$ cells/count), *t*-test. **h.** Endogenous co-immunoprecipitation (IP) of PcG proteins SUZ12 with EZH2 and RING1B with BMI1 in siScramble control (siC) or siSAFA (siS)-treated presenescent proliferating BJ fibroblasts or presenescent proliferating (PS) and RAS senescent (S) BJ fibroblasts. Immunoprecipitates were western-blotted either with EZH2 (upper panel) or BMI1 (lower panel). Asterix indicates IgG light chain and arrow BMI1. **i.** Presenescent proliferating BJ fibroblasts were treated with siScramble control (siC) or siSAFA (siS) for 3 days and whole cell lysates were analyzed by western blot using antibodies to $H3K27_{me3}$, -$H2AK119_{Ub1}$, -SAFA and -H2A.

Supplementary Figure 4

A.

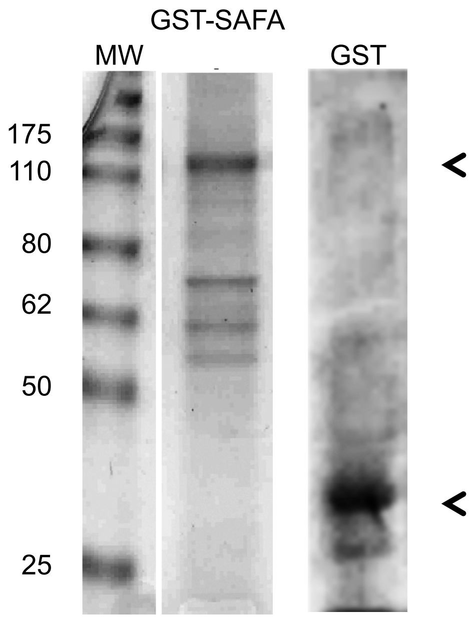

Supplementary Figure 4. SAFA and PRCs are negative co-regulators of pro-senescence factor *CDKN1A* and lncRNA-PANDA. Purification of recombinant GST-SAFA and GST from *E. coli* used in electrophoretic mobility shift assay of Figure 4C. Arrows indicate respective purified proteins that were used for EMSA in Figure 4C.

Supplementary Figure 5

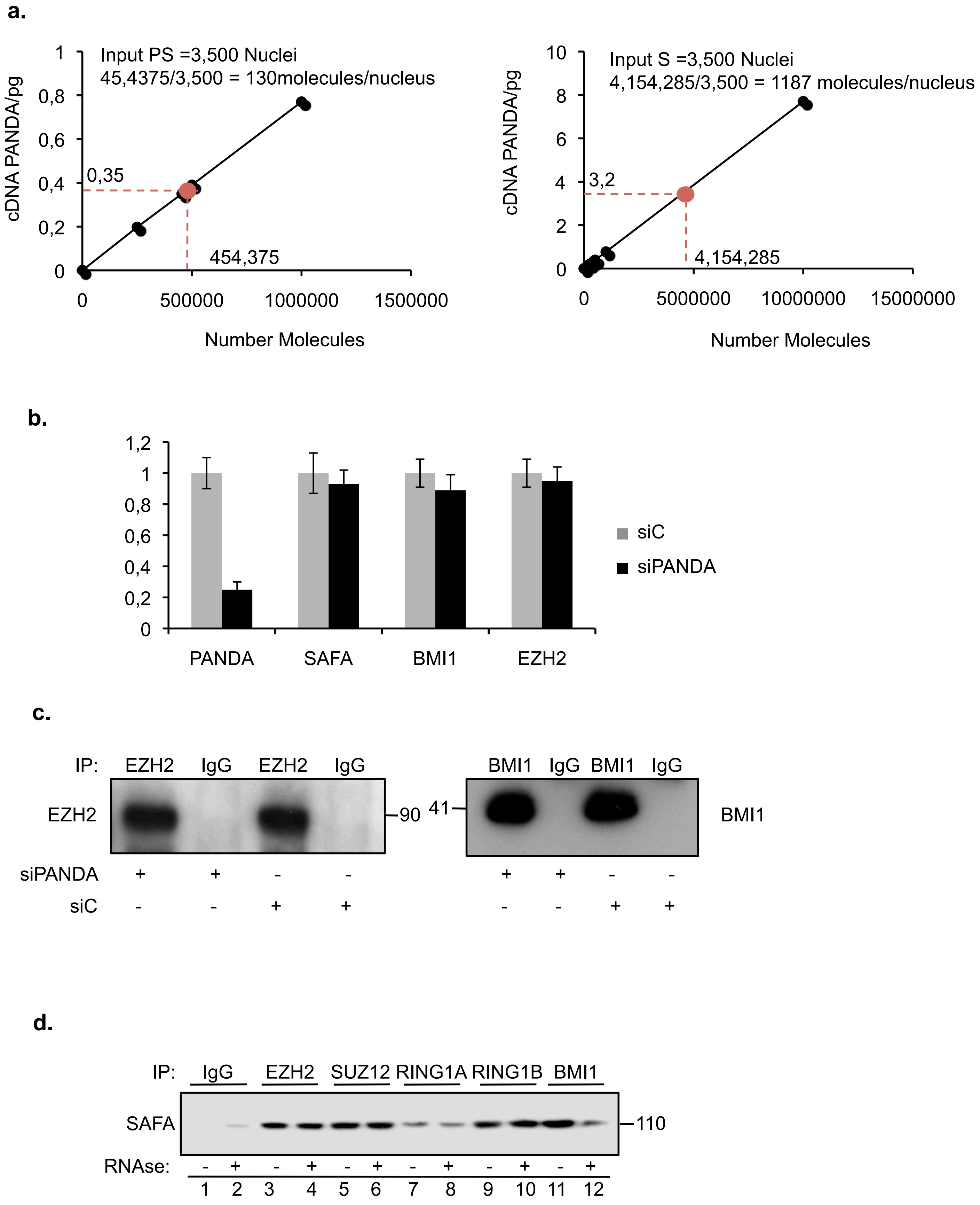

Supplementary Figure 5. PANDA binds to SAFA and BMI1 and is critical for their interaction. a. Titration curve used to determine number of PANDA molecules per nucleus. Red dotted lines signify PANDA molecules in presenescent proliferating (PS)(left graph) and RAS senescent (S)(right graph) cells. **b.** Presenescent proliferating BJ fibroblasts were treated with siScramble control (siC) or siPANDA for 3 days. qRT-PCR on total RNA prepared from respective samples for indicated genes. Error bars represent the s.d. of three independent experiments; $P \leq 0,05$, *t*-test. **c.** Efficacy of antibodies to EZH2 and BMI1 for native qRIP experiment of Figure 5E. **d.** Representative (n = 3) endogenous co-immunoprecipitation was performed in whole cell lysates treated with (+) or without (-) RNAse A between SAFA and PcG proteins EZH2, SUZ12, RING1A, RING1B and BMI1.

Supplementary Figure 6

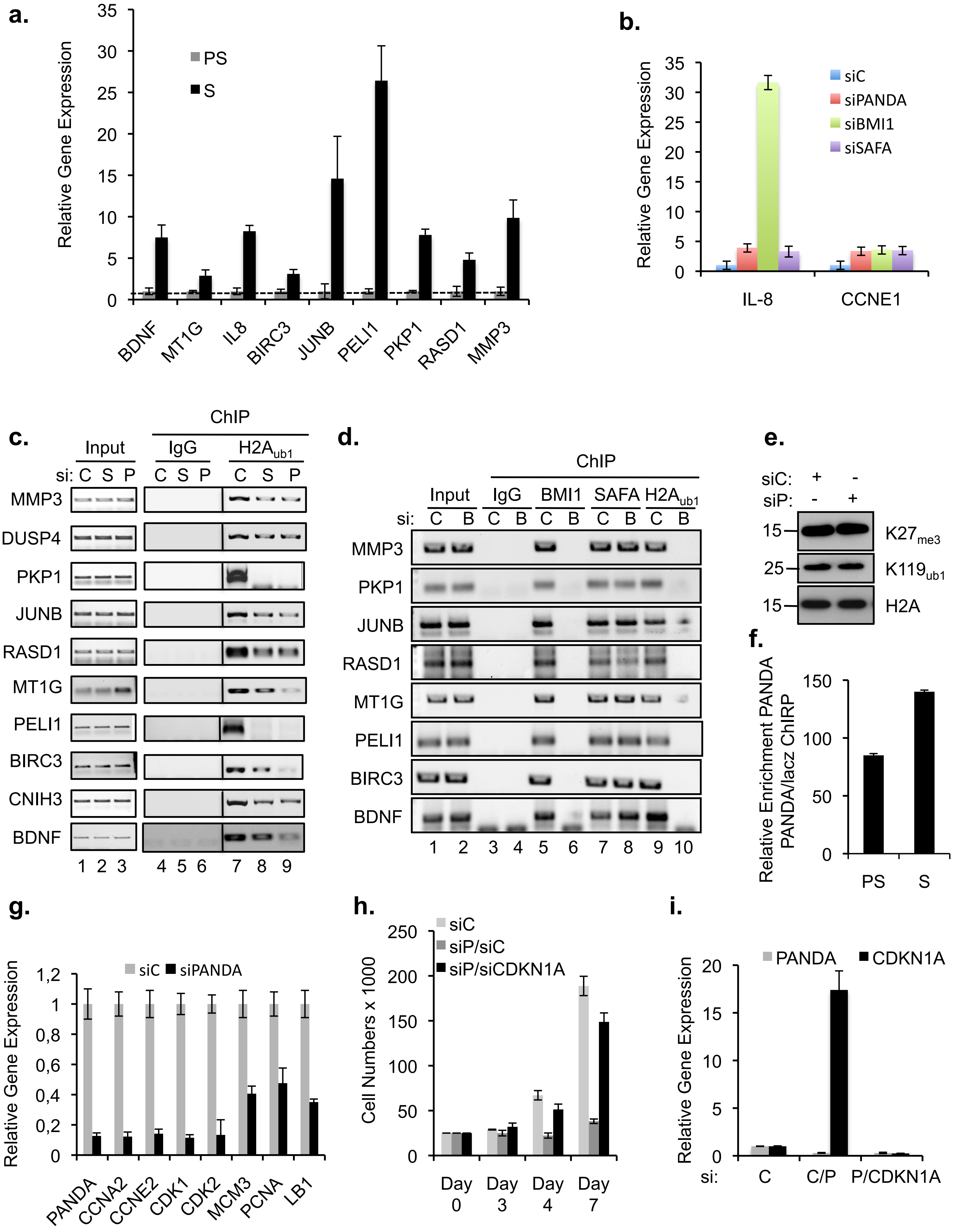

Supplementary Figure 6. PANDA regulates PRC target gene expression and senescence entry. **a.** qRT-PCR analysis of indiacted genes on total RNA prepared from presenescent proliferating (PS) and RAS senescent (S) BJ fibroblasts. Error bars represent the s.d. of three independent experiments; $P \leq 0,05$, *t*-test. **b.** Presenescent proliferating BJ fibroblasts were transiently transfected with indicated siRNA duplexes. qRT-PCR on total RNA prepared from respective samples at day 2 of siRNA treatment for indicated genes. Error bars represent the s.d. of three independent experiments; $P \leq 0,05$, *t*-test. **c.** IgG and $H2A_{Ub1}$ ($H2AK119_{Ub1}$) ChIP-PCR of indicated gene promoter regions in siScramble control (siC), siSAFA (siS) and siPANDA (siP)-treated presenescent proliferating BJ fibroblasts. **d.** IgG, BMI1, SAFA and $H2A_{Ub1}$ ($H2AK119_{Ub1}$) ChIP-PCR of indicated gene promoter regions in siScramble control (siC), and siBMI1 (siB)-treated presenescent proliferating BJ fibroblasts. Shown is a representative of three independent experiments (n = 3). **e.** Presenescent proliferating BJ fibroblasts were treated with siScramble control (siC) or siPANDA (siP) for 3 days and whole cell lysates were analyzed by Western blot using antibodies to $H3K27_{me3}$, $H2AK119_{Ub1}$ and H2A. **f.** qRT-PCR analysis for PANDA enrichment in ChIRP assay using biotinylated PANDA-specific antisense and lacZ control DNA probes in pre-senescent (PS) and RAS-senescent (S) BJ fibroblasts. Error bars represent the s.d. of three independent experiments (n = 3); $P \leq 0,05$), *t*-test. **g.** Presenescent proliferating BJ fibroblasts were transiently transfected with indicated siScramble control and siPANDA. qRT-PCR on total RNA prepared from respective samples at day 2 of siRNA treatment for indicated genes. Error bars represent the s.d. of three independent experiments (n=3); $P \leq 0,05$), *t*-test. **h-i.** CDKN1A depletion rescues siPANDA-induced senescence. **h.** Presenescent proliferating BJ fibroblasts were transiently transfected or co-transfectd either with siScramble control (siC), siPANDA pool (siP)/siC or siP/siCDKN1A for a duration of 7 days. Relative cell numbers at day 0, 2, 4 and 7 post siRNA-treatment are depicted. Error bars represent the s.d. of three independent experiments; $P \leq 0,05$), *t*-test. **i.** Relative *PANDA* and *CDKN1A* gene expression in samples of H as determined by qRT-PCR analysis. Error bars represent the s.d. of three independent experiments; $P \leq 0,05$), *t*-test.

Supplementary Figure 7

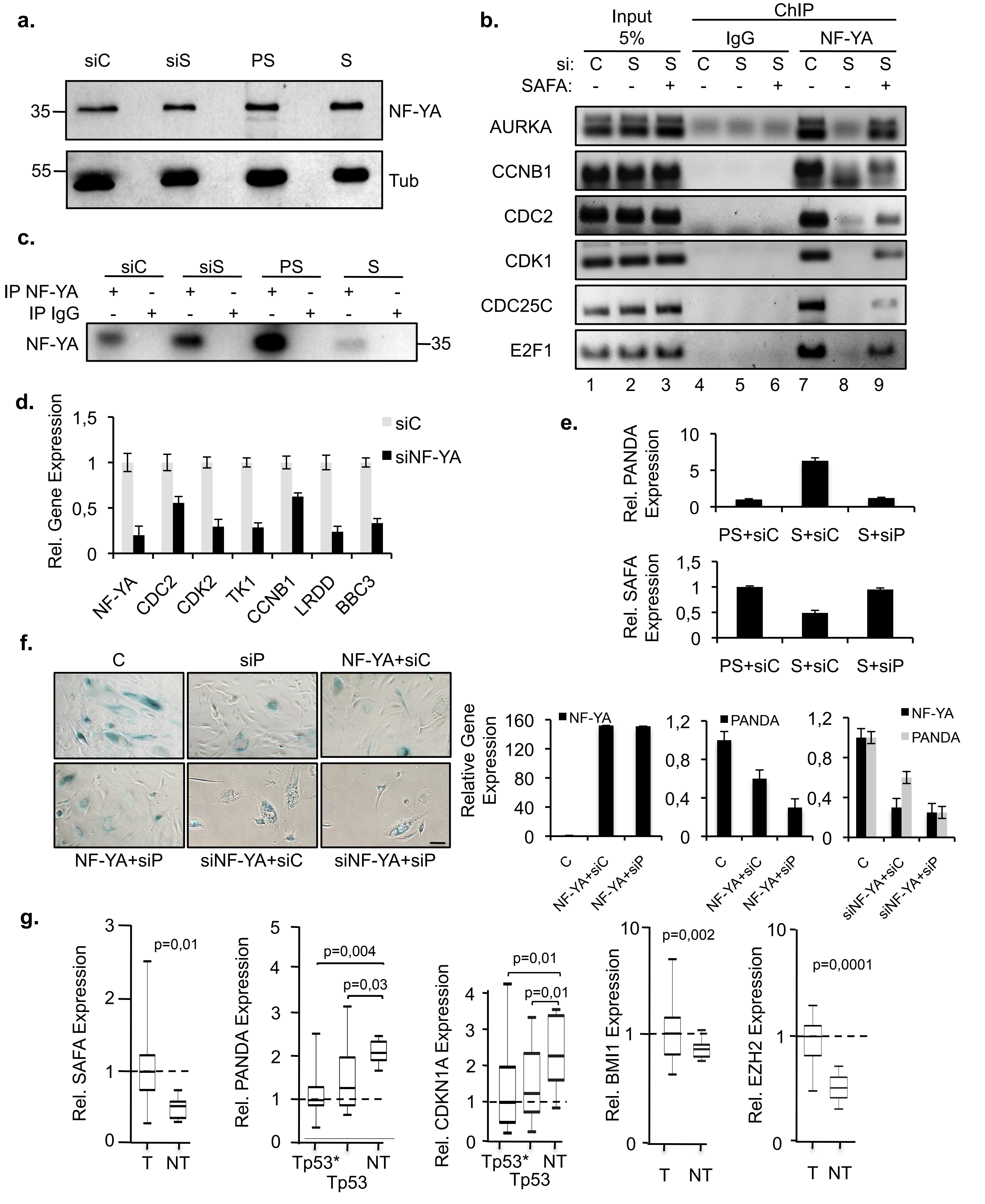

Supplementary Figure 7. PANDA regulates NF-Y:E2F-coregulated expression of pro-proliferation genes and senescence exit. a. Representative (n = 3) western blot analysis using antibodies to SAFA and Tubulin (Tub, loading control) of whole-cell-lysates prepared from siScramble control (siC) or siSAFA (siS)-treated presenescent proliferating BJ fibroblasts or presenescent proliferating (PS) and RAS senescent (S) BJ fibroblasts. **b.** IgG and NF-YA ChIP-PCR was performed in presenescent proliferating BJ fibroblasts treated for 3 days with siScramble control (siC) or siSAFA (siS) and overexpressing SAFA (+) or not (-). Shown is a representative of three independent experiments (n = 3). **c.** Immunoprecipitation (IP) of NF-YA in presenescent proliferating BJ fibroblasts treated with siScramble control (siC) or siSAFA (siS)- and presenescent proliferating (PS) or RAS senescent (S) BJ fibroblasts. Western blot with antibody to NF-YA. Shown is a representative of three independent experiments (n = 3). **d.** Presenescent proliferating BJ fibroblasts were treated with siScramble control (siC) or siNF-YA. qRT-PCR analysis for NF-YA target genes on total RNA prepared 3 days post treatment. Error bars represent the s.d. of three independent experiments; P ≤ 0,05), *t*-test. **e.** Presenescent proliferating (PS) and RAS senescent (S) BJ fibroblasts were treated with siScramble control (siC) or siPANDA (siP) for 3 days. qRT-PCR was performed for PANDA (left panel) or SAFA (right panel) on total RNA prepared from respective samples. Error bars represent the s.d. of three independent experiments; P ≤ 0,05, *t*-test. **f.** Representative photographs (scale bar 20 μm) of RAS senescent cells transfected with the indicated combinations. Note the decrease in SABG positive cells in NF-YA overexpressing (NF-YA) and siPANDA (siP) alone or together as well as diminished cell numbers indicative of ongoing cell death in siNF-YA treated cells. C is senescence cells treated either with siC or empty vector control. Also, shown are relative gene expression of NF-YA and PANDA compared to respective controls in these cell populations. **g.** SAFA, PANDA, CDKN1A, BMI1 and EZH2 expression levels in tumors (T)(n = 35) compared to non-tumors (NT)(n = 5). For PANDA and CDKN1A expression is also displayed for P53 mutant (*) vs. P53 wildtype tumors. Human primary hepatocelluar carcinomas (HCC) were derived from the fresh-frozen tissue bank of PP (see Supplementary Table S2 for details).

Supplementary Figure 8

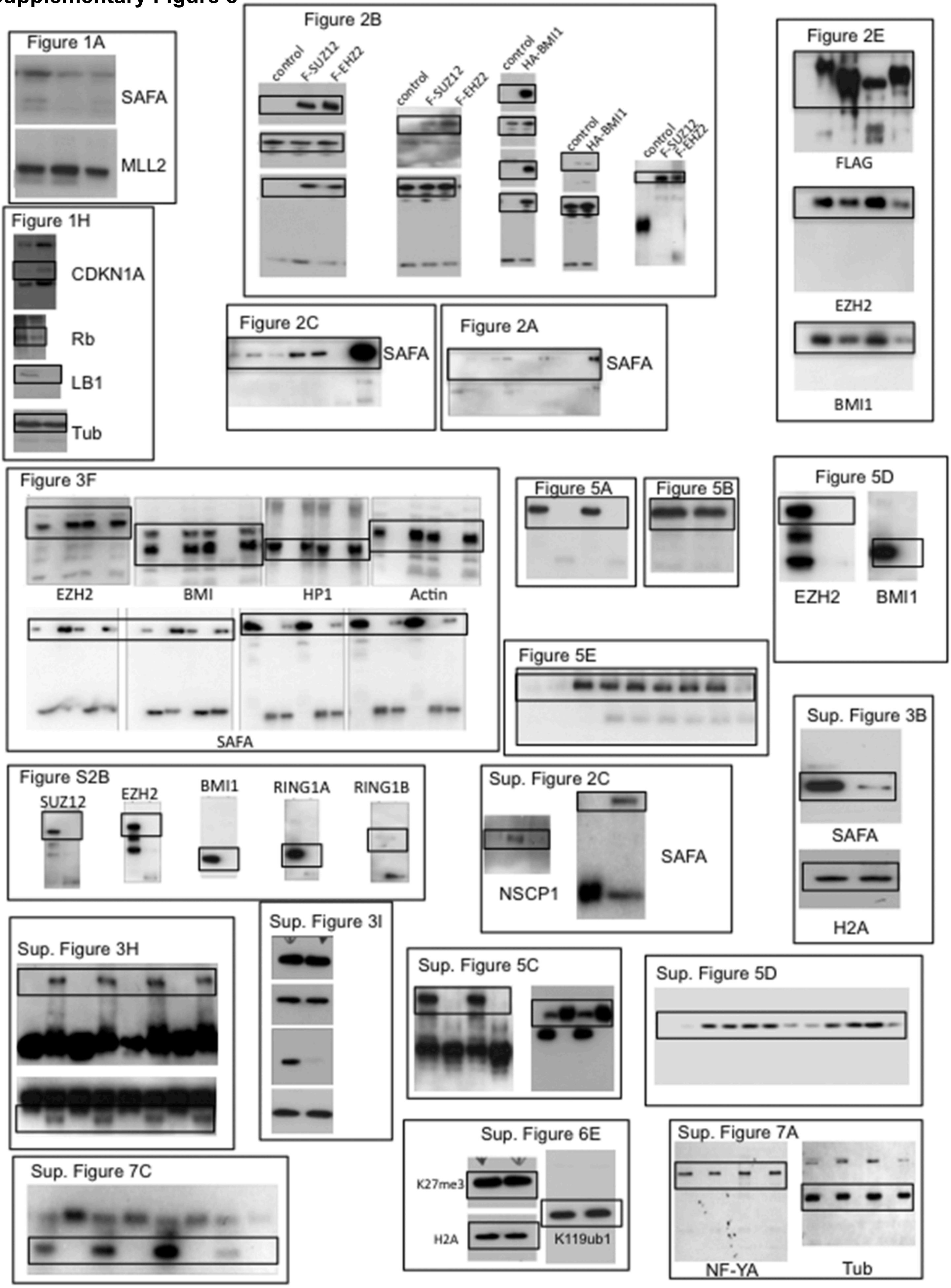

Supplementary Figure 8. Original scans of western blots.

Supplementary Table 1:

Genotype of p53, sex and risk factor in hepatocellular carcinoma (HCC) patient cohort used for meta-analysis in Supplementary Figure 7g.

**Sample ID**	**Sex/ Age**	**Risk Factor**	***p53* genotype**
28T	M41	HBV	*R249S*
30T	M47	HBV	*R110L*
31T	M43	HBV	*V157F*
33T	M22	HBV	*R249S*
35T	M60	HBV	*D184N*
79T	M49	HBV	*S121T*
81T	M34	HBV	del a 872-fs-344*
83T	M45	HBV	*Y326C*
87T	M46	HBV	*R335C+S336L*
88T	M68	HBV	*P152L*
90T	M59	HBV-HCV	*R309I*
92T	M41	HBV	*R249S*
96T	M30	HBV	*R249S*
97T	M52	HBV	*R249S*
99T	M60	HBV	*R267P*
102T	M54	HBV	*R249S*
105T	F40	HBV	*R249S*
134T	F40	HBV	*Q104H*
185T	M40	HBV	*R249S*
190T	M37	HBV	*R249S*
192T	M33	HBV	*E339**
193T	M66	HBV	*R110L*
2T	F31	HBV	wt
3T	M26	HBV	wt
27T	M39	HBV	wt
32T	M50	HBV	wt
34T	M43	HBV	wt
85T	M48	HBV	wt
94T	M50	HBV	wt
100T	M51	HBV	wt
186T	F44	HBV	wt
187T	M66	HBV-HCV	wt
188T	M63	HBV	wt
191T	M53	HBV	wt
207T	M40	cryptogenic	wt
Clinico-Biological features of patients (n=35) and *TP53* genotypes of corresponding primary liver cancers.			

Supplementary Table 2

qChIP-PCR primers used in this study:

DUSP4 FP: ATGGCTTACGATGGCACTG

DUSP4 RP: TTTAAGAAGGGCGCCGAAA

BMP2 FP: GGATCCCACGTCTATGCTATG

BMP2 RP: CAGGGTCTGGCCTCTTATTT

DLX2 FP: GTAATGCTCGCTTCTTAACCAATC

DLX2 RP: CCAGGGAAAGTGGTTTCAAATATAC

CNIH3 FP: CCAGGTGCTCTAAGATTCACTC

CNIH3 RP: GTCATCAAATCCAGAAAGCAAGG

P21 −511−645 FP: AGTGGACCTCAATTTCCTCATC

P21 −511−645 RP: TATTCCCACTGATCCCTCACTA

P21 −4269−4381 FP: CGAGATGATGCCAACCAGAT

P21 −4269−4381 RP: CCCATTGCCTGTTCCTCAA

MMP3 FP: GAGAGAAGAAGTAGGTTGACTTGG

MMP3 RP: AGCTATGTATGTACACTTTCCACTT

PKP1 FP: TGGTCTCTGCTATGACCTCAG

PKP1 RP: GGAGACCTCTCTCTAGGAAAGAAA

JUNB FP: CAATGGATTGTCAGTCCTCCTAC

JUNB RP: GGAGTCCACTGGGACAAATAC

RASD1 FP: CTGTCCCTCTGCGACTTC

RASD1 RP: ACTGGGTTACTTACTAGATTCCTATTC

MT1G FP: AGATGGGCCAAGTGCAAG

MT1G RP: GTGAGAGAAGCCGCACAC

PELI1 FP: CGGTCTTGTATTATGGTGTTGTTG

PELI RP: TCAGACCTCACTGGGTACTTAT

BIRC3 FP: CCCTACATTTCCTTTCACCTCTTAC

BIRC3 RP: GCTGCAGAAGTCCAGCATTTA

BDNF FP: TTCAGAAACCCTCACAGTCATC

BDNF RP: AGCATCTGCTGGCTAATTCA

WNT7B FP: CAACTCACACAACGCACATAA

WNT7B RP: GTGGTGTGTCTGTATGGTGT

AURKA FP: CTGCCTTGTGATTGGTTGAC

AURKA RP: CACCACTTCCGGGTTCTTA
CCNB1 FP: GCCACGAACAGGCCAATAA
CCNB1 RP: AGAAACCAACAGCCGTTCC
CDK1 FP: CTCCGCTGACTAGAAACAGTAG
CDK1 RP: GCGCGAAAGAAAGAGGAAAG
CDC25C FP: GCCAGAGCAGGATAGTGTTT
CDC25C RP: ACAGAAAGGGTGTGGAGATTG

Semi-quantitative ChIP-PCR primers used in this study:
DUSP4 FP: CGAGGGCACCGGTACCCGCCGGGTCTCTCC
DUSP4 RP: GGACTAGGGTGAGCACAAGCCTTGAGCGC
BMP2 FP: CCTCCTATTCCGGGGAAACAGAAGCGTGG
BMP2 RP: GCCGCGGCCGAACACCTCCCCCTTCG
DLX2 FP: GTAATGCTCGCTTCTTAACCAATCCGCAGGG
DLX2 RP: TTGCAATGATAGGGTAGAACAGGGTCCTAAA
CNIH3 FP: TGCAGGAATGTTGGGGCATAGTAGAAAG
CNIH3 RP: GACAAGTCATCAAATCCAGAAAGCAAGGAAA
P21 +37−342 FP: GCTCATTCTAACAGTGCTGTG
P21 +37−342 RP: CAAGGAACTGACTTCGGCAG
P21 −324−676 FP: CCCGGAAGCATGTGACAATC
P21 −324−676 RP: CAGCACTGTTAGAATGAGCC
P21 −677−981 FP: GGAGGCAAAAGTCCTGTGTTC
P21 −677−981 RP: GGAAGGAGGGAATTGGAGAG
P21 −964−1340 FP: CTGAGCAGCCTGAGATGTCAG
P21 −964−1340 RP: CACAGGACTTTTGCCTCCTG
P21 −1335−1688 FP: GAAATGCCTGAAAGCAGAGG
P21 −1335−1688 RP: GCTCAGAGTCTGGAAATCTC
P21 −2029−2478 FP: CACCACTGAGCCTTCCTCAC
P21 −2029−2478 RP: CTGACTCCCAGCACACACTC
P21 −3141−3558 FP: GACATAGCAGGTGTGATGACC
P21 −3141−3558 RP: GTATTCAGGTGGCTGAGGTG
P21 −4149−4525 FP: CGCGGTGCTTGGTCTCTATG
P21 −4149−4525 RP: CCTTTCCCAACAAACAAGGGG
MMP3 FP: GTAGGATGATACAACCTACTTTACTAATTTC

MMP3 RP: GTATAGACTATAGCTATGTATGTACACTTTCCAC

PKP1 FP: GGAGCAAGAGCTATGGTTCTGCCATCAGCC

PKP1 RP: CAGGGAGACGCCCCTCTGTCCTGTTCTGC

JUNB FP: CCAATGGATTGTCAGTCCTCCTACCCCTCTC

JUNB RP: GGAAGCGCGTGTCCTTGTAAACAGCGGCCACG

RASD1 FP: GAGCAGTCCAGGGTCGGGCTGAGGCTGGAGG

RASD1 RP: GAGGACTGCAATATACGGTCCGCGCAAGCAC

MT1G FP: GTGGAAGGCGGAGTGGAGCCCAACAGCCA

MT1G RP: GAAGGCCTGGCAGGGTGAGAGAAGCCGCAC

PELI1 FP: CTCCCACCGTTACCATGGTCACCGACACAC

PELI RP: GTTTGTGGAAGATAAATTACGCAGCAGGGAAG

BIRC3 FP: CACATACCCCTACATTTCCTTTCACCTCTTAC

BIRC3 RP: GATAACAACCCATATAGAGAGGACGATCATTG

BDNF FP: AGGAGTAGTTCTAGATTTGCCAGGCTCC

BDNF RP: TGGTATGTACCAGCTGTCCCAGTCTTAG

WNT7B FP: ACACAGCACATACCAGAAATCACACAAACA

WNT7B RP: GTGGTGTGGTATGGGTGCATGGTGTATG

AURKA FP: CTTACTGGCACGTTCAAAGGTTAGT

AURKA RP: GGAGTTAAACCCTCTAGCTAGAAAGC

CCNB1 FP: CGATCGCCCTGGAAACGCATTC

CCNB1 RP: CCCAGCAGAAACCAACAGCCGTTC

CDC2 FP: AAACAGTAGGACGACAC

CDC2 RP: CGCTCAATTTCCAAGAGCCAGCT

CDK1 FP: GAACTGTGCCAATGCTGGGA

CDK1 RP: GCAGTTTCAAACTCACCGCG

CDC25C FP: GAATGGACATCACTAGTAAGGCGCG

CDC25C RP: GCAGGCGTTGACCATTCAAACCTTC

E2F1 FP; GGCTACAGGTTGAGGGTCACG

E2F1 RP:GAGCGCCGCCACAATTGGCT

TK1 FP: AGACACATCCATCATGGCGTCTACA

TK1 RP: GAAGTTCACGAACCCGAGTACTCT

Supplementary Table 3

qRT-PCR primers:

DUSP4 FP: CCTGGCAGCCATCCCACCCCCGGTTCCCC

DUSP4 RP: GCTGATGCCCAGGGCGTCCAGCATGTCTCTC

WNT7B FP: CCAACATCATCTGCAACAAGATTCCTG

WNT7B RP: CGAAGACGGTCTTCTCGCCGAGGGCAGAG

CDKN1A FP: TCAGAGGAGGCGCCATGT

CDKN1A RP: TGTCCACTGGGCCGAAGA

BMP2 FP: GGAGCTGGGCCGCAGGAAGTTCGCGGC

BMP2 RP: TGGTCTGGGGCGGGTGAGCCCGGCTGA

DLX2 FP: CTCCAGCACGTACCACCAGCACCAGCAG

DLX2 RP: CTGGTAGGAACCCATGTGCGCGATGGGCGAG

CNIH3 FP: CCCCATAGACCAGTGCAATCCTGTTC

CNIH3 RP: AGGGACATTCAGCCCCAGCGTGAGCCAC

MMP3 FP: CCTGCTTTGTCCTTTGATGC

MMP3 RP: TGAGTCAATCCCTGGAAAGTC

PKP1 FP: CTCAGACAGCAAGGTTTCGATAG

PKP1 RP: GTGAACTTTCCCAAATACTGCAC

JUNB FP: ATGGAACAGCCCTTCTACCACG

JUNB RP: AGGCTCGGTTTCAGGAGTTTG

RASD1 FP: GAACTGCTATCGCATGGTCATCCTCGGCT

RASD1 RP: GGAGTAGAACTTGCGGTGGAAGTCCTC

MT1G FP: TTCCCTTCTCGCTTGGGAACTCTA

MT1G RP: GCTCTTCTTGCAGGAGGTGCATTT

PELI1 FP: TGTTCCTCTGTGGCTTGGATGTGA

PELI1 RP: TTGTTCACCAGCCAACTGATGTGC

BIRC3 FP: ATGAAGCCAGGTGTGGTGGTATGT

BIRC3 RP: TCAGTATGCTGCCCAGGATGGATT

BDNF FP: ATTGGCTGGCGATTCATAAG

BDNF RP: GTTTCCCTTCTGGTCATGGA

PANDA FP: TGCACACATTTAACCCGAAG

PANDA RP: CCCCAAAGCTACATCTATGACA

HPRT FP: GCTGGTGAAAAGGACCTCT

HPRT RP: CACAGGACTAGAACACCTGC
IL8-FP: GGCACAAACTTTCAGAGACAG
IL8-RP: ACACAGAGCTGCAGAAATCAGG

siRNA SAFA:
S102781002: CTGGCCGTGGTAGTTACTCAA
S102780540: AGGATATTATTGAATACCCAA

Supplementary Table 4

Probes for chromatin-isolation by RNA purification

PANDA:

GAGGATGCCTCTCTTCAAAC

ACAGGTGTCATAGATGTAGC

CCAGGTTGGTAACACTGATG

TCTGCTGCAAATCTCAGTTT

GGAATTACCTTTGACAGTGG

CCAACCAGATTTGCCGAAAT

GGTGAAGCTCTATGGAACTG

AGGTGGATCCCTGTAGAGAT

TGAACATATCTGGACCACCT

ACTAAAATACAAACATTGGG

LacZ:

TAAATGTGAGCGAGTAACAACC

TGCCATAAAGAAACTGTTACCC

GAAGGATCGACAGATTTGATCC

ATTTAATCAGCGACTGATCCAC

GTCAGCAGTTGTTTTTTATCGC
